# Absolute Structure Determination and Kv1.5 Ion Channel Inhibition Activities of New Debromoaplysiatoxin Analogues

**DOI:** 10.3390/md19110630

**Published:** 2021-11-11

**Authors:** Sicheng Shen, Weiping Wang, Zijun Chen, Huihui Zhang, Yuchun Yang, Xiaoliang Wang, Peng Fu, Bingnan Han

**Affiliations:** 1 Department of Development Technology of Marine Resources, College of Life Sciences and Medicine, Zhejiang Sci-Tech University, Hangzhou 310018, China; 201920201063@mails.zstu.edu.cn (S.S.); 202020801010@mails.zstu.edu.cn (Z.C.); 201820201062@mails.zstu.edu.cn (H.Z.); 2019339902026@mails.zstu.edu.cn (Y.Y.); 2 Institute of Materia Medica, Chinese Academy of Medical Sciences and Peking Union Medical College, Beijing 100730, China; wangwp@imm.ac.cn (W.W.); wangxl@imm.ac.cn (X.W.); 3 Key Laboratory of Marine Drugs, Ministry of Education of China, School of Medicine and Pharmacy, Ocean University of China, Qingdao 266003, China

**Keywords:** marine cyanobacterium, debromoaplysiatoxin analogues, absolute configuration, Kv1.5 inhibitory activity, brine shrimp toxicity

## Abstract

Potassium channel Kv1.5 has been considered a key target for new treatments of atrial tachyarrhythmias, with few side effects. Four new debromoaplysiatoxin analogues with a 6/6/12 fused ring system were isolated from marine cyanobacterium *Lyngbya* sp. Their planar structures were elucidated by HRESIMS, 1D and 2D NMR. The absolute configuration of oscillatoxin J (**1**) was determined by single-crystal X-ray diffraction, and the absolute configurations of oscillatoxin K (**2**), oscillatoxin L (**3**) and oscillatoxin M (**4**) were confirmed on the basis of GIAO NMR shift calculation followed by DP4 analysis. The current study confirmed the absolute configuration of the pivotal chiral positions (7S, 9S, 10S, 11R, 12S, 15S, 29R and 30R) at traditional ATXs with 6/12/6 tricyclic ring system. Compound **1**, **2** and **4** exhibited blocking activities against Kv1.5 with IC_50_ values of 2.61 ± 0.91 µM, 3.86 ± 1.03 µM and 3.79 ± 1.01 µM, respectively. However, compound **3** exhibited a minimum effect on Kv1.5 at 10 µM. Furthermore, all of these new debromoaplysiatoxin analogs displayed no apparent activity in a brine shrimp toxicity assay.

## 1. Introduction

Many marine creatures have developed various ways to mark themselves as predators or preys over the course of evolution, and marine toxins have often played an important role in these relationships [1]. A variety of marine toxins block or activate ion channels [2,3,4,5,6]. For instance, conotoxins were discovered as mammalian voltage-gated potassium channel (Kv) 1 blockers [7], shellfish toxin saxitoxin (STXs) exhibited tetrodotoxin-sensitive voltage-gated sodium channels (Navs) blocking activity [8] and ciguatoxins, a kind of polyether toxins, acted as sodium channel activators [9]. Moreover, many cyanotoxins were identified as modulators of the sodium/ potassium channels [10,11,12,13,14].

Voltage-gated K^+^ channels (Kv) are membrane-inserted K^+^ selective protein complexes [15]. Kv channels are important for various physiological and pathophysiological processes [16,17]. The Shaker-related Kv1 family consisting of subtypes (Kv1.1–Kv1.8) present in most mammalian peripheral tissues such as cardiovascular, nervous and the immune system, and many of them have been identified as potential targets for a variety of marine toxins [18,19,20]. Dalazatide (ShK-186), targeting the Kv1.3 ion channel [21], has completed phase 1 clinical trials for the treatment of autoimmune diseases [22]. Gambierol, a marine polycyclic ether toxin produced by the dinoflagellate *Gambierdiscus toxicus* with IC_50_ of 34.5 ± 1.5 nM aganist Kv1.2 channel, might be deemed as a lead compound in further studies of the treatment of pathogenic conditions [23]. Two toxins identified from the venom of *Bunodosoma caissarum*, BcsTx1 and BcsTx2, displayed the highest affinity for Kv1.6 with IC_50_ of 1.31 ± 0.20 nM and 7.76 ± 1.90 nM, respectively [24].

**Figure 1:**
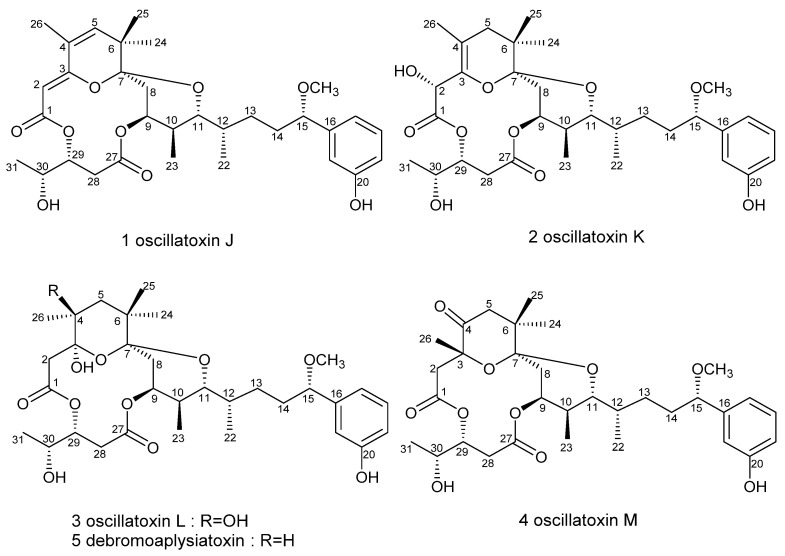
Structures of identified debromoaplysiatoxin analogues in this study: new compounds **1**–**4** and reported Compound **5**.

The aplysiatoxins (ATXs) and their related analogues (oscillatoxins and nhatrangins) are distinct polyketide classes of marine toxins isolated from several cyanobacterial species, including *Oscillatoria nigro-viridis, Schizothrix calcicola* and *Lyngbya majuscula* [25,26,27,28]. In our previous studies, we have pre-screened many ATXs and debromoaplysiatoxin analogues (DATs) for inhibitory activity on the shaker-related subfamily of voltage-gated channels (Kv1.1–Kv1.5), and the results indicated that some analogues presented selective and strong blocking effects on potassium channel Kv1.5 [29]. The ultrarapid activating delayed rectifier K^+^ current (IKur) carried by the Kv1.5 channel is the main repolarization current in human atria but has no effect in the ventricle [30,31]. Therefore, Kv1.5 has become a significant molecular target for the treatment of atrial tachyarrhythmias with minimum side effects. In order to find additional novel Kv1.5 inhibitors, our team has isolated four new debromoaplysiatoxin analogues (Figure 1), oscillatoxin J–M (**1**–**4**) from the cyanobacterium *Lyngbya* sp. The planar structures of these compounds were elucidated by analysis of MS and NMR data, and the absolute configurations were determined by single-crystal X-ray diffraction combined with gauge invariant atomic orbital (GIAO) NMR shift calculation followed by DP4 analysis. DP4 is a probability analysis method based on the errors in each ^13^C or ^1^H chemical shift in the GIAO NMR calculation. The bioactivity results implicated that these four new DAT analogues and DAT exhibited differential Kv1.5 blocking activities and brine shrimp toxicities in correlation with the existence of structural functionality change.

## 2. Results and Discussion

### 2.1. Structure Elucidation

**Figure 2:**
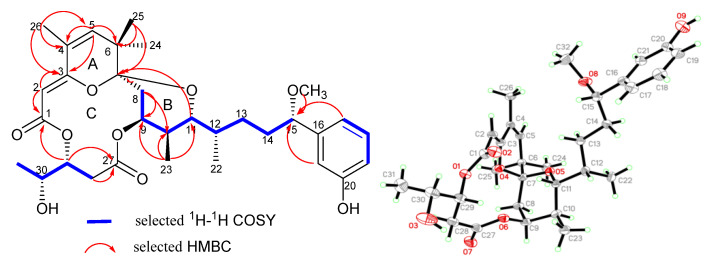
Key 2D correlations and X-ray structure of **1**.

Oscillatoxin J (**1**), obtained as white solid ([α${]}_{D}^{25}$ + 1.0 (*c* = 0.3, MeCN); UV (MeOH) _λmax_ (log ε) 194 (3.91), 268 (2.47) nm (Figure S2.1.9)), was assigned to a molecular formula of C_32_H_44_O_9_ with 11 degrees of unsaturation, as established by HRESIMS at *m/z* 595.2886 [M + Na]^+^ (calcd for C_32_H_44_O_9_Na, 595.2883). Its ^13^C and DEPT NMR spectra exhibited 32 carbon signals, attributed to one methoxy, six methyls, four methylenes, thirteen methines and eight quaternary carbons (Table 1). The ^1^H NMR spectrum and the ^13^C NMR spectrum of **1** bore a close resemblance to that of oscillatoxin B1 [32], except a double bond between C-4 and C-5 instead of a hydroxyl group on C-4. The HMBC correlations of H-5 to C-3, C-4, C-6 and C-7 and H_3_-26 to C-3, C-4 and C-5 strongly supported this assignment. Hence, the planar structure of **1** was established. Furthermore, the structure of **1** was proved by X-ray analysis (Figure 2).

X-ray diffraction analysis of **1** with Cu Kα radiation established the absolute configuration of **1** to be 7S, 9S, 10S, 11R, 12S, 15S, 29R and 30R with an absolute structure parameter of 0.06(18), consistent with the sequential correlations of H-9/H-10, H-10/H_3_-22 and H-11/H_3_-23 in the ROESY spectrum (Supplementary Information Figure S2.1.7).

Previously, only 19,21-dibromoaplysiatoxin [33] and neo-debromoaplysiatoxin A [34] were reported with the successful obtainment of crystal structures. In this study, the determination of absolute structure of oscillatoxin J by using X-ray diffraction analysis further confirmed the pivotal chiral positions (C-7, C-9/C-10/C-11/C-12, C-15 and C-29/C-30) of DATs with a 6/12/6 tricyclic ring system, which provided a reference for the determination of stereochemistry of the series of compounds.

**Figure 3:**
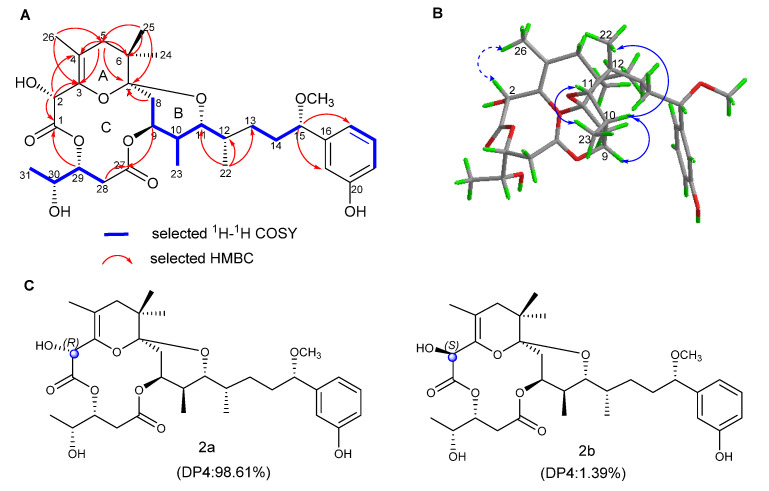
(**A**) Key 2D correlations of **2**. (**B**) Key NOESY correlations of **2** (solid lines: α-orientation; dashed lines: β-orientation). (**C**) DP4 analysis results for **2**.

Oscillatoxin K (**2**) was isolated as a white solid ([α${]}_{D}^{25}$ + 64.4 (*c* = 0.36, MeCN); UV (MeOH) _λmax_ (log ε) 198 (2.71), 275 (0.55) nm (Supplementary Information Figure S2.2.9)) with a molecular formula C_32_H_46_O_10_, as determined from the HRESIMS at *m/z* 613.2980 [M + Na]^+^ (calcd for C_32_H_46_O_10_Na, 613.2989). Thirty-two carbon resonances can be observed in ^13^C and DEPT spectra, including eight quaternary carbons, twelve methines, five methylenes and seven methyls (Figure 3). Its NMR data were similar to 2-hydorxyanhydroaplysiatoxin, except for the absence of a bromine atom on C-17 [28]. The detailed ^1^H and ^13^C NMR signal assignments and connectivity were determined from a combination of ^1^H-^1^H COSY, HSQC and HMBC data (Supplementary Information Figures S2.2.1–S2.2.6). 

**Figure 4:**
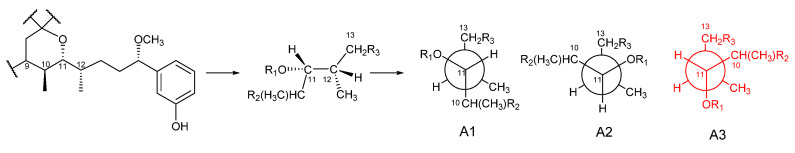
Newman projection analysis of the energy equivalent isomer.

The relative configuration of compound **2** was ascertained by detailed Nuclear analysis Overhauser Effect Spectroscopy (NOESY) spectrum, the coupling constants, NMR analysis and biogenetically related configuration inference of compound **2**. The NOE correlations between H-9 and H-8a and small couplings of H-9 to H-8b (J_8b,9_ =2.8 Hz) proved that H-9 showed an equatorial position on ring B. H-10 and H-11 were determined as axial orientation by the large coupling constant (J = 10.1 Hz) of H-10/H-11. On the basis of NOE correlation of H-11/H-12, Newman’s projection analysis of energy equivalent isomers using nuclear coupling constant information and steric hindrance (Figure 4) was performed. The analysis suggested a gauche conformer of H-11/H-12, which is possessed by oscillatoxin J–M (1-4). For compound **2**, the three large groups (–OR1, –CH_2_R3 and –CH(CH_3_) R2) in model A2 were extremely close in space, causing a large steric hindrance; hence, model A2 was also eliminated. The ^1^H-^1^H coupling constant (1.8Hz) between H-11 and H-12 indicated that there was a gauche relationship between these two protons; thus, model A1 was excluded. H-11 and H-12 were oriented in the same plane, which was confirmed by remaining model A3. The NOESY spectrum correlations between H-9/H-10, H_3_-23/H-11/H-12 and H-10/H_3_-22 proved that these protons were had the same orientations. In addition, taking notice of the structural similarities of oscillatoxin J–M (**1**–**4**), these four compounds are likely to have a common biosynthetic origin [26]. The coupling constants (J = 11.9 Hz) of H-29/H-28a and (J = 2.1Hz) of H-29/H-28b, in keeping with those of aplysiatoxins, indicated the syn relationship between H-29 and H-30. The chemical shifts of H-15 and its coupling constants (J = 6.4 Hz) were similar to those of aplysiatoxins [33]. Simulated and experimental ^13^C NMR chemical shifts of 2a and 2b were used for DP4 probability analysis. The calculations were performed by using the density functional theory (DFT) as carried out in the Gaussian 09 [35]. The statistical results indicated the structural equivalence of **2** to 2a (98.61% probability). In summary, the absolute configuration of **2** was established as 2R, 7S, 9S, 10S, 11R, 12S, 15S, 29R and 30R.

**Figure 5:**
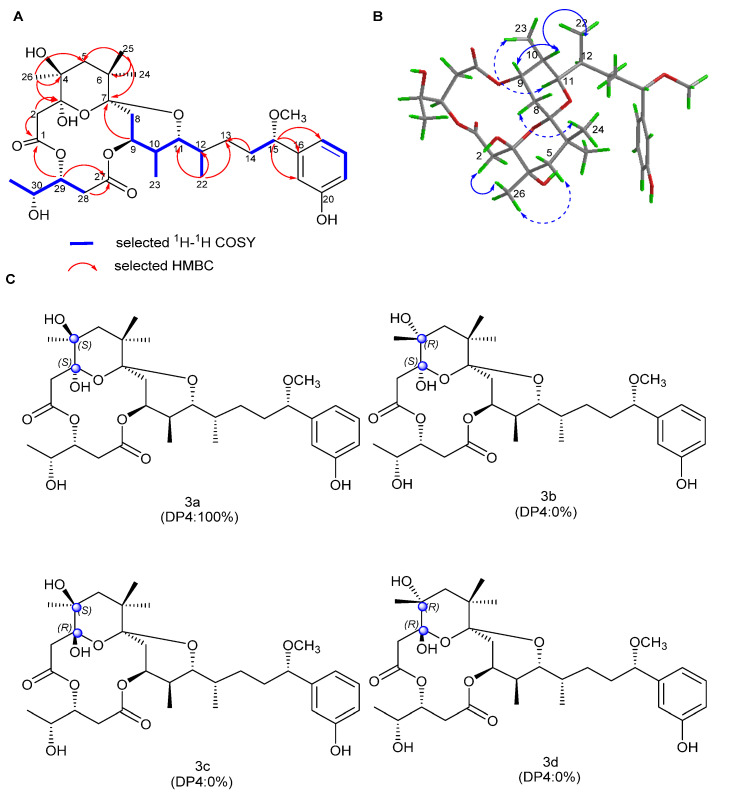
(**A**) Key 2D correlations of **3**. (**B**) Key NOESY correlations of **3** (solid lines: α-orientation; dashed lines: β-orientation). (**C**) DP4 analysis results for **3**.

Oscillatoxin L (**3**) was obtained as a white solid ([α${]}_{D}^{25}$ + 35 (*c* = 0.1, MeCN); UV (MeOH) _λmax_ (log ε) 197 (2.57), 275 (0.78) nm (Supplementary Information Figure S2.3.9)). It has a molecular formula of C_32_H_48_O_11_, with nine degrees of unsaturation, as assigned by HR-ESI-MS data (*m/z* 631.3087 [M + Na]^+^, calculated for C_32_H_48_O_11_Na, 631.3094). The interpretation of the 1D and 2D NMR spectra indicated that the planar structure of **3** closely agreed with debromoaplysiatoxin except for a hydroxyl on C-4 in **3**. The HMBC correlations of H_3_-26 to C-3, C-4 and C-5 and H-2 to C-1 and C-3 strongly supported this connection. By comparing the ^1^H chemical shift and coupling constant of **3** with that of DAT **3,** it was observed that they have the same stereochemical properties as DAT, except for C-3 and C-4 [36]. The DP4 analysis was again applied to the simulated ^13^C NMR chemical shifts of the four possible epimers 3a–3d (Figure 5). The results showed that the correct structure of compound 3 is the epimer 3a, with 100% probability (Figure 5 and Table S1.4.8., Supplementary Materials Information). Hence, the absolute configuration of **3** was determined to be 3S, 4S, 7S, 9S, 10S, 11R, 12S, 15S, 29R and 30R (Figure 5).

**Figure 6:**
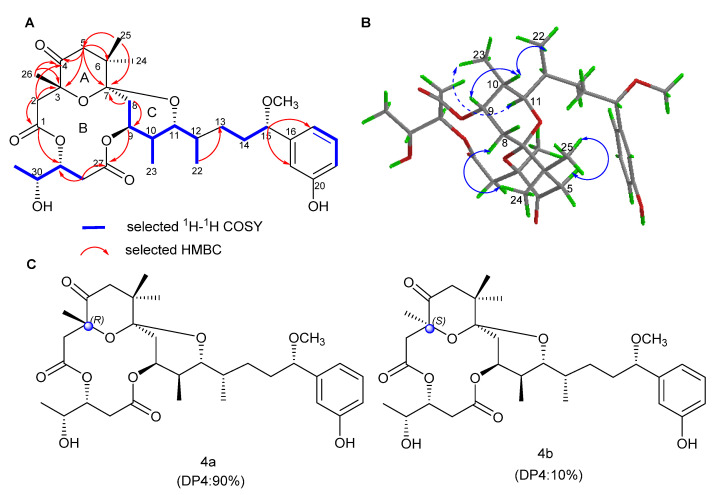
(**A**) Key 2D correlations of **4**. (**B**) Key NOESY correlations of **4** (solid lines: α-orientation; dashed lines: β-orientation). (**C**) DP4 analysis results for **4**.

Oscillatoxin M (**4**) was isolated as a white solid ([α${]}_{D}^{25}$ + 35 (*c* = 0.3, MeCN); UV (MeOH) _λmax_ (log ε) 196 (2.52), 275 (0.59) nm (Figure S2.4.9)). The molecular formula of C_32_H_46_O_10_ with 10 degrees of unsaturation was inferred from HRESIMS data at *m/z* 613.2994 [M + Na]^+^ (calcd for C_32_H_46_O_10_Na, 613.2989). The inspection of spectral data showed that the planar structure of oscillatoxin M (**4**) was identical to that of debromoaplysiatoxin (**5**), with the exception that C-3 had a methyl group instead of a hydroxyl group, and C-4 had a ketone carbonyl group instead of a methyl group. The HMBC correlations of H-5 to C-3, C-4 and C-6; H_3_-26 to C-3 and C-4; and H-2 to C-1, C-3 and C-4 strongly support this assignment. The relative configuration of H-9/H-10/H-11/H-12, H-15 and H-29/H-30 in compound **4** was determined in accordance with that of compound **2**. Furthermore, the results of DP4 statistical analysis using the ^13^C NMR chemical shift values proved that the correct structure for **4** is the epimer 4a (Figure 6 and Supplementary Information Table S1.4.11.). Finally, the absolute configuration of **4** was established as 3R, 7S, 9S, 10S, 11R, 12S, 15S, 29R and 30R.

### 2.2. Bioactivities

#### 2.2.1. Inhibitory Activities against Kv1.5

**Figure 7:**
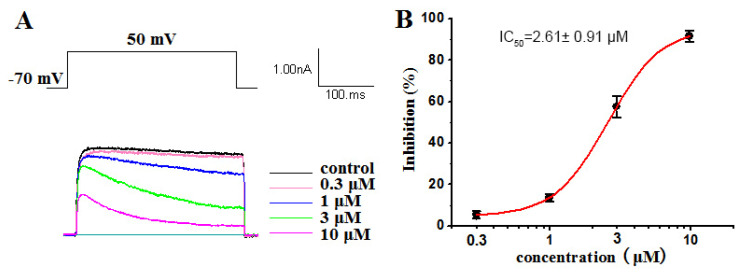
(**A**) Kv1.5 currents were evoked by a 300 ms depolarizing pulse from –50 mV to 50 mV in 20 mV increments from a holding potential of –70 mV in the absence and presence of 0.3 μM, 1 μM, 3 μM and 10 μM oscillatoxin J (**1**). The current amplitudes were measured at the end of the 300 ms pulse at 50 mV. (**B**) Concentration–inhibition curve expressed in %. The abscissa represents the concentration, and the ordinate represents the percentage of Kv1.5 current that is blocked at different concentrations of oscillatoxin J (**1**). Data points represent mean ± SEM of 3 to 5 measurements, and the inhibitory effect showed an IC_50_ value of 2.61 ± 0.91 µM.

**Figure 8:**
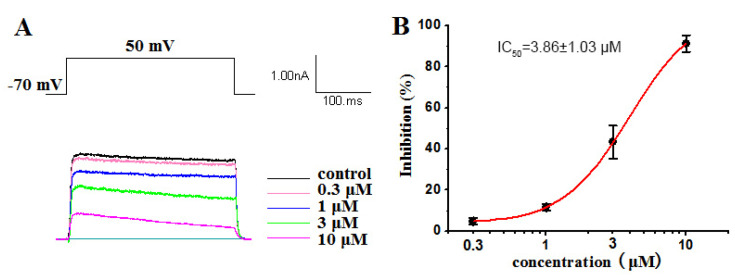
(**A**) Kv1.5 currents were evoked by a 300 ms depolarizing pulse from –50 mV to 50 mV in 20 mV increments from a holding potential of –70 mV in the absence and presence of 0.3 μM, 1 μM, 3 μM and 10 μM oscillatoxin K (**2**). The current amplitudes were measured at the end of the 300 ms pulse at 50 mV. (**B**) Concentration–inhibition curve expressed in %. The abscissa represents the concentration, and the ordinate represents the percentage of Kv1.5 current that is blocked at different concentrations of oscillatoxin K (**2**). Data points represent mean ± SEM of 3 to 5 measurements, and the inhibitory effect showed an IC_50_ value of 3.86 ± 1.03 µM.

**Figure 9:**
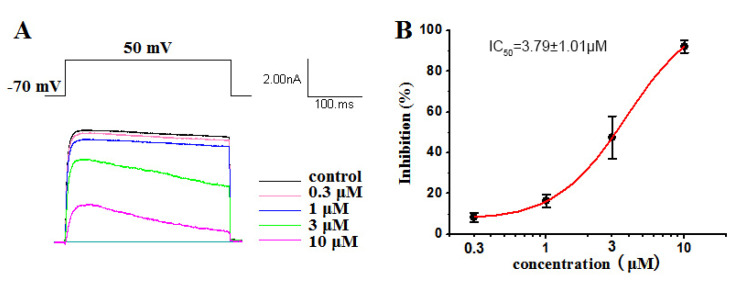
Kv1.5 currents were evoked by a 300 ms depolarizing pulse from –50 mV to 50 mV in 20 mV increments from a holding potential of –70 mV in the absence and presence of 0.3 μM, 1 μM, 3 μM and 10 μM oscillatoxin M (**4**). The current amplitudes were measured at the end of the 300 ms pulse at 50 mV. (**B**) Concentration–inhibition curve expressed in %. The abscissa represents the concentration, and the ordinate represents the percentage of Kv1.5 current that is blocked at different concentrations of oscillatoxin M (**4**). Data points represent mean ± SEM of 3 to 5 measurements, and the inhibitory effect showed an IC_50_ value of 3.79 ± 1.01 µM.

The Kv1.5 (ultra-fast-delay rectifier potassium channel) mediation of ultra-rapid delayed rectifier K^+^ current (IKur) is the main current in the repolarization process of atrial action potentials. Our previous research has highlighted the capability of ATXs as ion channel blockers [34,37]. Some compounds have blocked Kv1.5 with certain selectivity [29]. In this work, a dose–response study was conducted on compounds **1**–**4** to evaluate their inhibitory activity against Kv1.5. Our results showed that compounds **1**, **2** and **4** had significant inhibitory effects on Kv1.5, with IC_50_ values of 2.61 ± 0.91 µM (Figure 7), 3.86 ± 1.03 µM (Figure 8) and 3.79 ± 1.01 µM (Figure 9), respectively. Compound 1, 2 and 4 all voltage dependently inhibited the Kv1.5 current (Supplementary Materials 1.3). The inhibition was stronger at 50 mV than other tested potentials. The voltage dependence supported the fact that three compounds preferentially affected the open state of the Kv1.5 channels. However, compound **3**, oscillatoxin L, is structurally almost identical to DAT (IC_50_ = 1.28 ± 0.08 µM), except having an additional hydroxy motif on adjacent carbon C-4, exhibiting minimum effects on the modulation of Kv1.5 at 10 µM (Figure S1.3.1). Aplysiatoxin and its derivatives are activators of protein kinase C (PKC), which has been well researched [38]. In a previous study, we have demonstrated DAT strongly upregulating the expression of phosphor PKC*δ* in human hepatocellular carcinomas (HepG2) at 10 µM [29] and proposed the potential mechanism for DATs modulating the Kv channel by activating protein kinase C [39]. The results might indicate that the simple modifications of the functionalities on A ring system in DAT structure may affect either the interaction of PKC or the blocking site of the Kv1.5 ion channel. Consequently, determining the mechanism of Kv1.5 inhibition activity of DAT analogues will be our ongoing project.

#### 2.2.2. Toxicity of Brine Shrimp

**Figure 10:**
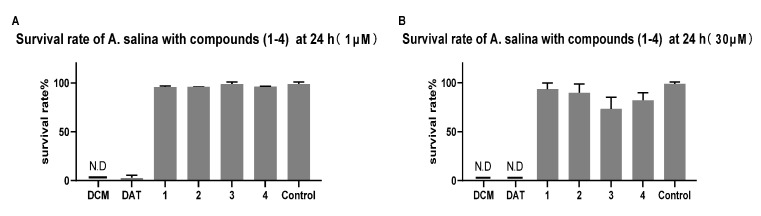
Dose effect of compounds **1**–**4** to *Artemia salina* (*A. salina*). *A. salina* was treated with indicated concentration (1 µM and 30 µM) of dichloromethane (DCM), debromoaplysiatoxin (DAT) and compounds **1**–**4** for 24 h. (**A**) The percentage of *A. salina* compounds **1**–**4** in 1 µM; (**B**) the percentage of *A. salina* with compounds **1**–**4** in 30 µM. N.D: the life of brine shrimp not detected. Data were analyzed by GraphPad prism (Table S1.3.2.1).

In order to understand the toxicity effect of such compounds, brine shrimp *Artemia salina* (*A. salina*) was used as a model organism. The investigation of brine shrimp toxicity of debromoaplysiatoxin (DAT) and four DAT analogs (oscillatoxin J, oscillatoxin K, oscillatoxin L and oscillatoxin M) isolated from marine cyanobacterium *Lyngbya* sp., was conducted. When DAT concentration was as low as 0.1 µM, the survival of *Artemia salina* (*A. salina*) began to be affected (Table S1.3.2.1). Compounds **1**, **2**, **3** and **4** had no apparent effect at 30 µM. As shown in Figure 10, compared to other tested derivatives, debromoaplysiatoxin was the most toxic compound (IC_50_ value = 0.34 ± 0.036 µM) (Figure S1.3.2.1). The current results with a previous study [37] indicated that the 3-hydroxy group at DAT seemed to play an important role in determining higher toxicity. However, the specific mechanism of toxic action of these compounds remains unclear, and further studies are needed.

## 3. Materials and Methods

### 3.1. General Experimental Procedure

UV was acquired on a UV/EV300 spectrometer (Thermo Scientific, Waltham, MA, USA), and IR spectra were obtained on a Nicolet iS20 instrument (Thermo Fisher Scientific, MA, USA). Optical rotation data were performed by a Jasco P-2000 polarimeter (Jasco, Hachiojishi, Tokyo, Japan). ^1^H and ^13^C NMR spectra were acquired on an Agilent 600 MHz spectrometer (Agilent Technologies, Santa Clara, CA, USA), with CDCl_3_ (*δ*H 7.26 and *δ*C 77.16) as the solvent and internal standard. HRESIMS data were collected with a Bruker micrOTOF-Q II mass spectrometer (Bruker Daltonik GmbH, Bremen, Germany). A Shimadzu LC-16 series instrument (Shimadzu, Kyoto, Japan) was equipped with C-18 column (5 µm, 10 mm × 250 mm, YMC, Kyoto, Japan) and an SPD-M20A diode array detector (Shimadzu, Kyoto, Japan) for high-performance liquid chromatography (HPLC) analysis. For column chromatography, Silica gel 60 (200–300 mesh; Yantai, China) and octadecylsilyl (ODS) (50 µm, YMC, Kyoto, Japan) were used. A silica gel 60 F254 plate (Merck, Darmstadt, Germany) was used for analytical thin-layer chromatography.

### 3.2. Material

Cyanobacterium *Lyngbya* sp. was obtained from the harbor of Sanya, Hainan province, China, in November 2016. The sample was identified by Prof. Bing-Nan Han (Zhejiang Sci-Tech University, Zhejiang, China). After morphological and molecular identification, a voucher specimen (voucher number: BNH-201606; gene bank accession numbers: MH636576) has been well deposited in Zhejiang Sci-Tech University.

### 3.3. Extraction and Isolation

Cyanobacterium freeze-dried powder (150 g) was extracted with CH_2_Cl_2_/MeOH (1:1, *v*/*v*). The obtained extract was dissolved in 1 L of MeOH/H_2_O (9:1, *v*/*v*) and extracted with CH_2_Cl_2_ (3 × 1 L) in order to obtain the CH_2_Cl_2_ extract (20 g), which was subjected to vacuum liquid chromatography (VLC) over silica gel to obtain seven subfractions (F. A–G). The separation conditions of the gradient used gradients of PE/EtOAc (5:1, 2:1, 1:1, 1:2, 1:5, 0:1, *v*/*v*). F.B, (2000 mg) was further separated by reversed-phase octadecylsilyl silica (ODS) (UV detection at 254 nm, flow rate 20 mL/min, 10%–100% MeCN/H_2_O and 180 min) in order to acquire 17 subfractions (F.B.1–17). Subsequently, the subfraction F.B.7 (231 mg) was purified by semi-preparative HPLC (Shimadzu silgreen C-18, 70% MeCN/H_2_O, 2.0 mL/min and UV detection at 190 nm) to collect oscillatoxin J (8 mg), oscillatoxin K (15.2 mg), oscillatoxin L (3.7 mg) and oscillatoxin M (25.4 mg) (Supplementary Materials 1.2).

### 3.4. Ion Channel Inhibitory Experiment

#### 3.4.1. Cell Culture

For culturing mouse L cells lacking thymidine kinase (LTK) cells stably expressing human Kv1.5 channels (LTK/Kv1.5), 10% fetal bovine serum (FBS), 10,000 U/ml penicillin G and 10 mg/ml streptomycin were added into Dulbecco’s Modified Eagle Media (DMEM). LTK/Kv1.5 cells were cultured in a 37 °C humidified incubator set to 5% CO_2_. The amount of 100 μg/ml Genetic (G418) was added into the culture media to select transgenic cells. When the cells grew to 75–85% confluence, they were passaged for subsequent electrophysiological records (Supplementary Materials 1.3.1.1).

#### 3.4.2. Electrophysiology

LTK cells cultured for at least 24 h could be used for currents recording. For Kv1.5 potassium current recording, the recording micropipettes were pulled with a P97 microelectrode puller (Sutter, CA, USA) with a resistance of about 3 MΩ when filling with internal solution containing the following: KCl 140 mM, MgCl_2_ 1 mM, EGTA 5 mM, HEPES 10 mM and MgATP 1 mM (pH was adjusted to 7.25 by KOH). The bath solution contained the following: NaCl 137 mM, KCl 5.4 mM, CaCl_2_ 1.8 mM, MgCl_2_ 1 mM, Glucose 10 mM and HEPES 10 mM (pH was adjusted to 7.4 by NaOH). Kv1.5 currents were recorded at room temperature (22–24 °C) by PulseMaster (Version 2.65, Heka, Lambrecht, Germany) via an EPC-10 USB amplifier (Heka, Lambrecht, Germany). In order to reduce recording errors, cells with seal resistance above 1 GΩ and series resistance that was fully compensated above 80% were used. Leak compensation was used to compensate the leak current and to subtract the capacitive artifacts (Supplementary Materials 1.3.1.2).

### 3.5. Brine Shrimp Toxicity Assay

Brine shrimp *A. salina* is an important model organism for ecosystems, and because of its high sensitivity and easy availability, it can be used in laboratory settings to study toxic effects and to provide safe results. *A. salina* or brine shrimp cysts were cultivated in 3.2% of saline water. After aeration with normal saline, cysts were placed at temperature for 24 h and then cultured. For toxicity screening, hatched larvae were collected and introduced in saline water. Add 0.9% brine and 30 larvae with good activity to each well to produce a 96-well test culture plate. New compounds of 0.1 µM, 1 µM, 10 µM and 30 µM and DAT were added to the experimental culture plate, respectively. Dimethyl sulfoxide (Aladdin, Shanghai, China) and dichloromethane (Aladdin, Shanghai, China) were added as blank control and positive control. The survival rate of *A. salina* was calculated after 24 h treatment at 25 °C. 

## 4. Conclusions

In conclusion, four new debromoaplysiatoxin analogues, oscillatoxin J–M (**1**–**4**), were isolated from the cyanobacterium *Lyngbya* sp. The structures of the new compounds were characterized by 1D and 2D NMR and MS data. ATXs easily underwent structural rearrangement to form new structures with new functionality and new stereochemistry because of the presence of some unstable functional groups such as hemiacetal and ketal, etc. However, the current study confirmed the absolute configuration of pivotal chiral positions (7S, 9S, 10S, 11R, 12S, 15S, 29R and 30R) at traditional ATXs with 6/12/6 tricyclic ring system of compounds **1**–**4** via X-ray diffraction and GIAO NMR shift calculation followed by DP4 analysis. Compound **1**, **2** and **4** showed inhibitory activities against Kv1.5 with IC_50_ value of 2.61 ± 0.91 µM, 3.86 ± 1.03 µM and 3.79 ± 1.01 µM, respectively. Compound **3** exhibited no apparent Kv1.5 inhibition activity at 10 µM. Discovery of new DAT analogs may highlight some insightful structures and activity relationships for developing strong, effective and safe Kv1.5 inhibitors in the future. Our future work will focus on characterizing the selectivity of these compounds for Kv1.5 and how they inhibit Kv1.5 channel. 

## Figures and Tables

**Table 1:** ^1^H (600 MHz) and ^13^C (150 MHz) NMR data for compounds **1**–**4** in CDCl_3_ (δ in ppm; J in Hz).

Pos.	1		2		3		4	
	*δ*_H_ (*J* in Hz)	*δ*_C_	*δ*_H_ (*J* in Hz)	*δ*_C_	*δ*_H_ (*J* in Hz)	*δ*_C_	*δ*_H_ (*J* in Hz)	*δ*_C_
1		169.0		171.1		174.2		166.2
2	5.04, s	95.0	5.04, s	70.2	a 3.00, d (11.7)	40.0	3.36, d (13.3)	46.3
					b 2.45, d (11.7)		3.68, d (13.3)	
3		159.3		140.2		101.1		86.3
4		124.3		110.3		72.6		203.2
5	5.67, s	140.8	a 2.25, m	39.9	a 3.00, d (11.7)	43.4	a 1.43, m	44.9
			b 1.33, d (16.9)		b 2.45, d (11.7)		b 2.65, d (12.7)	
6		39.8		35.7		37.7		47.1
7		102.5		99.9		102.0		108.7
8	a 2.56, d (14.9)	30.7	a 1.64, m	30.4	a 2.13, dd (15.0, 2.8)	32.8	a 2.34, dd (14.6, 3.3)	31.6
	b 1.65, m		b 2.27, m (2.8)		b 1.53, dd (14.9, 3.6)		b 1.41(m)	
9	4.8, m	73.6	4.84, q (2.9)	74.3	4.82, q (3.1)	73.3	4.75, q (2.8)	74.2
10	1.67, m	34.4	1.60, ddd (10.1, 6.8, 2.8)	34.1	1.14, m	33.9	1.56, m	34.1
11	3.80, d (10.5)	71.7	3.54, dd (10.6, 1.8)	72.0	3.65, dd (10.8, 1.9)	72.6	3.63, dd (10.6, 1.9)	73.7
12	1.47, m	33.1	1.25, m	33.5	1.59, dt (6.9, 3.3)	33.5	1.32, m	33.8
13	a 1.36, d (6.6)	30.5	a 1.36, m	30.3	a 1.39, td (13.7, 12.1, 6.6)	30.0	a 1.25, m	30.0
	b 1.33, d (6.6)		b 1.28, m		b 1.27, d (14.6)		b 1.19, m	
14	a 1.68, m	35.9	a 1.83, m	36.0	a 1.95, m	36.1	a 1.89, m	36.2
	b 1.53, m		b 1.62, m		b 1.5, m		b 1.52, m	
15	3.93, dd (8.4, 4.8)	84.9	4.01, t (6.8)	85.0	4.06, t (6.9)	84.8	4.07, dd (7.7, 5.6)	84.8
16		144.1		144.4		144.1		144.1
17	6.8, m	117.8	6.85, dt (7.6, 1.2)	118.5	6.87, m	118.2	6.90, dt (7.6, 1.2)	118.3
18	7.20, d (7.7)	129.7	7.18, t (7.8)	129.6	7.22, t (7.8)	129.9	7.20, t (7.8)	129.8
19	6.71, dt (7.9, 1,1)	115.2	6.72, ddd (8.1, 2.6, 1.0)	114.5	6.88, m	114.7	6.84, t (1.9)	114.9
20		156.8		156.2		155.9		155.9
21	6.8, m	114.0	6.89, t (2.0)	114.4	6.76, ddd (8.0, 2.5, 1.0)	114.8	6.75, m	114.6
22	0.77, overlap	12.8	0.75, d (6.5)	12.0	0.63, d (6.9)	13.8	0.71, d (6.7)	11.3
23	0.78, overlap	13.4	0.73, d (6.9)	13.4	0.73, d (6.9)	12.2	0.78, d (6.8)	13.8
24	0.99, s	23.6	0.8, s	22.8	0.86, s	27.1	0.97, s	23.6
25	1.05, s	23.5	0.9, s	24.4	1.12, s	26.5	1.00, s	25.6
26	1.68, m	17.6	1.66, s	16.9	1.20, s	18.1	1.47, s	27.2
27		170.7		169.8		169.9		170.1
28	a 2.88, dd (14.9, 5.6)	36.6	a 2.96, dd (17.9, 11.9)	36.9	a 2.80, m	35.4	a 2.96, dd (18.7, 11.2)	35.6
	b 2.63, dd (14.9, 4.9)		b 2.70, dd (17.9, 2.1)		b 2.79, m		b 2.70, dd (18.7, 1.3)	
29	5.14, d (5.2)	75.0	5.42, ddd (11.9, 5.0, 2.1)	74.0	5.3, dt (9.8, 5.0)	74.6	5.35, dd (11.4, 4.6)	74.0
30	4.27, p (6.3)	68.6	3.82, m	68.9	4.02, m	67.9	3.81, m	68.7
31	1.27, q (6.7)	18.9	1.23, d (6.4)	19.9	1.21, d (5.1)	26.4	1.15, d (6.4)	19.1
15-OCH_3_	3.2, s	56.9	3.23, s	56.7	3.24, s	56.9	3.26, s	56.8

NMR data of debromoaplysiatoxin (DAT) in Table S3 of Supplementary Materials.

## Data Availability

Data are contained within the article or Supplementary Materials.

## Supplementary Materials

The following are available online at https://www.mdpi.com/article/10.3390/md19110630/s1, experimental details; Figure S1.1.: Identification of cyanobacterium; Figure S1.3.1.1.– S1.3.1.4.: Ion channel experiment; Table S1.3.2.1.: Brine shrimp cytotoxicity assay; Tables S1.4.1.–S1.4.11.: NMR calculation and followed by DP4 analysis; Figures S2.1.–S2.4.: 1D and 2D NMR, HRESIMS, UV and IR spectra of compounds **1**–**4**.
