# Supplementary material for: Absolute Structure Determination and Kv1.5 Ion Channel Inhibition Activities of New Debromoaplysiatoxin Analogues
Source: Mar Drugs. 2021 Nov 11;19(11):630. doi: 10.3390/md19110630 (PMC8622842; doi:10.3390/md19110630)
Supplement: Supplementary file 1 [file marinedrugs-19-00630-s001.zip › marinedrugs-1403605-supplementary.pdf]

Supplementary Materials: Absolute Structure Determination and Kv1.5 Ion Channel Inhibition Activities of New Debromoaplysiatoxin Analogues

Sicheng Shen [1], Weiping Wang [2], Zijun Chen [1], Huihui Zhang [1], Yuchun Yang [1], Xiaoliang Wang [2], Peng Fu [3,*] and Binghan Han [1,*]

1 Department of Development Technology of Marine Resources, College of Life Sciences and Medicine,Zhejiang Sci-Tech University, Hangzhou 310018, China; 201920201063@mails.zstu.edu.cn (S.-C.S.); 202020801010@mails.zstu.edu.cn (Z.-J.C.); 201820201062@mails.zstu.edu.cn (H.-H.Z.); 2019339902026@mails.zstu.edu.cn (Y.-C.Y.)

2 Institute of Materia Medica, Chinese Academy of Medical Sciences and Peking Union Medical College, Beijing 100730, China, wangwp@imm.ac.cn (W.-P.W.); wangxl@imm.ac.cn (X.-L.W.)

3 Key Laboratory of Marine Drugs, Ministry of Education of China, School of Medicine and Pharmacy, Ocean University of China, Qingdao 266003, China

* Correspondence: fupeng@ouc.edu.cn (P.F.); hanbingnan@zstu.edu.cn (B.-N.H.); Tel.: +86-0532-82031836; Tel.: +86-571-8684-3303

Contents

1. Experimental Details 4
1.2. Extraction and Isolation 5
1.3. Bioassays 6
1.3.1. Ion Channel Experiment 6
1.3.1.1. Cell culture 6
1.3.1.2. Electrophysiology 6
1.3.1.3. Data analysis 6
1.3.2. Brine Shrimp Cytotoxicity Assay 8
1.4. Methods for NMR Calculation of oscillatoxin K-M (**2-4**) 9
Table S1.4.1.DFT-optimized structures and thermodynamic parameters for low-energy conformers of **2a** 10
Table S1.4.2. DFT-optimized structures and thermodynamic parameters for low-energy conformers of **2b** 10
Table S1.4.3. The calculated ^{13}C NMR data for **2.** 11
Table S1.4.4. DFT-optimized structures and thermodynamic parameters for low-energy conformers of **3a**. 12
Table S1.4.5. DFT-optimized structures and thermodynamic parameters for low-energy conformers of **3b.** 12
Table S1.4.6. DFT-optimized structures and thermodynamic parameters for low-energy conformers of **3c**. 13
Table S1.4.7. DFT-optimized structures and thermodynamic parameters for low-energy conformers of **3d**. 13
Table S1.4.8. The calculated ^{13}C NMR data for **3.** 15
Table S1.4.9. DFT-optimized structures and thermodynamic parameters for low-energy conformers of **4a**. 17
Table S1.4.10. DFT-optimized structures and thermodynamic parameters for low-energy conformers of **4b**. 17
Table S1.4.11. The calculated ^{13}C NMR data for **4.** 18
2. Figures 19
2.1 Spectra Data of compound **1.** 19
Figure S2.1.1 ^{1}H NMR spectrum of compound **1** in $CDCl_3$. 19
Figure S2.1.2 ^{13}C NMR spectrum of compound **1** in $CDCl_3$. 20
Figure S2.1.3 DEPT spectrum of compound **1** in $CDCl_3$. 20
Figure S2.1.4 HSQC spectrum of compound **1** in $CDCl_3$. 20
Figure S2.1.5 ^{1}H-^{1}H COSY spectrum of compound **1** in $CDCl_3$. 21
Figure S2.1.6 HMBC spectrum of compound **1** in $CDCl_3$. 21
Figure S2.1.7 ROESY spectrum of compound **1** in $CDCl_3$. 22
Figure S2.1.8 HRESIMS spectrum of compound **1** in MeOH. 22
Figure S2.1.9 UV spectrum of **1** in MeOH. 23
Figure S2.1.10 IR spectrum of **1**. 23
2.2 Spectra Data of compound **2**. 24
Figure S2.2.1 ^{1}H NMR spectrum of compound **2** in $CDCl_3$. 24
Figure S2.2.2 ^{13}C NMR spectrum of compound **2** in $CDCl_3$. 24

Figure S2.2.3 DEPT spectrum of compound **2** in $CDCl_3$....25
Figure S2.2.4 HSQC spectrum of compound **2** in $CDCl_3$....25
Figure S2.2.5 ^1H-^1H COSY spectrum of compound **2** in $CDCl_3$. 25
Figure S2.2.6 HMBC spectrum of compound **2** in $CDCl_3$....26
Figure S2.2.7 NOESY spectrum of compound **2** in $CDCl_3$. 26
Figure S2.2.8 HRESIMS spectrum of compound **2** in MeOH. 27
Figure S2.2.9 UV spectrum of **2** in MeOH. 27
Figure S2.2.10 IR spectrum of **2**....27
2.3 Spectra Data of compound **3**....28
Figure S2.3.1 ^1H NMR spectrum of compound **3** in $CDCl_3$....28
Figure S2.3.2 ^{13}C NMR spectrum of compound **3** in $CDCl_3$. 29
Figure S2.3.3 DEPT spectrum of compound **3** in $CDCl_3$....29
Figure S2.3.4 HSQC spectrum of compound **3** in $CDCl_3$....30
Figure S2.3.5 ^1H-^1H COSY spectrum of compound **3** in $CDCl_3$. 30
Figure S2.3.6 HMBC spectrum of compound **3** in $CDCl_3$....31
Figure S2.3.7 NOESY spectrum of compound **3** in $CDCl_3$. 31
Figure S2.3.8 HRESIMS spectrum of compound **3** in MeOH. 32
Figure S2.3.9 UV spectrum of **3** in MeOH. 32
Figure S2.3.10 IR spectrum of **3**....32
2.4 Spectra Data of compound **4**....33
Figure S2.4.1 ^1H NMR spectrum of compound **4** in $CDCl_3$....33
Figure S2.4.2 ^{13}C NMR spectrum of compound **4** in $CDCl_3$. 34
Figure S2.4.3 DEPT spectrum of compound **4** in $CDCl_3$....34
Figure S2.4.4 HSQC spectrum of compound **4** in $CDCl_3$....35
Figure S2.4.5 ^1H-^1H COSY spectrum of compound **4** in $CDCl_3$. 35
Figure S2.4.6 HMBC spectrum of compound **4** in $CDCl_3$....36
Figure S2.4.7. NOESY spectrum of compound **4** in $CDCl_3$. 36
Figure S2.4.8 HRESIMS spectrum of compound **4** in MeOH. 37
Figure S2.4.9 UV spectrum of **4** in MeOH. 37
Figure S2.4.10 IR spectrum of **4**....37
3. NMR Data for debromoaplysiatoxin 38

1. Experimental Details

1.1. Morphological and Molecular Identification of Cyanobacterium

The cyanobacterium strain used in this study were collected from Harbor of Hainan Sanya, China, Named as cyanobacterium HN. Colonies of cyanobacterium HN appeared as dark red, brown, or black tufts ranging from 15 to 25 cm in length and grew attached to sea rock and surface of the sea.

Filament width, cell width, and cell length of cyanobacterium HN were measured on the compound light microscope (Zeiss, Germany) with a 20× objective and 10× ocular lens with a calibrated optical micrometer. Filaments were long, of indeterminate length, 55–65 μm wide, formed by a uniseriate row of discoid cells encased in a firm, colorless, hyaline sheath which, when old, became yellowed and distinctly lamellated. Cells were discoid, 6–8 μm long, 30–40 μm broad, with rounded end cells without calyptra. Cell contents were finely granular without prominent granular inclusions (Figure S1.1A).

16S rDNA was used to characterize the identity of cyanobacterium samples. Total cyanobacterium genomic DNA from lyophilized samples was extracted by using TianGen Plant Genomic DNA Kit (TIANGEN Biotech Co., Ltd., Beijing, China) according to the manufacturer's instructions. Three PCR primer sets, CYA106F (5′-TACGGCTACCTTGTTAACGCGTGA-3′)/781R (5′-GACTACTGGGGTATC-TAATCCCATT-3′), 27F (5′-AGAGTTTGATCCTGGCTCAG-3′)/809R (5′-GC-TTCGGCACGGCTCGGGTCGATA-3′) and MSR2F (5′-CGGTAATACGGGG-GAGGCAA-3′)/2R (5′-CCAACATCTCACGACACGAG-3′), were used for amplifying 16S rDNA. PCR reactions were performed in a BIO-RAD Cycler C1000, according to the following profile: 5 min at 95 °C and 35 cycles of 30 s at 95°C, 1 min at 58 °C for CYA106F/781R, 30 s at 50 °C for 27F/809R or MSR2F/2R, and 1 min at 72 °C, followed by 10 min at 72 °C. The products were analyzed by electrophoresis in 0.7% (w/v) agarose gels electrophoresis. 16S rDNA sequences of other cyanobacterial taxa were acquired from NCBI GenBank and EzBioCloud databases and aligned by using MUSCLE implemented in MEGA7.0, and the phylogenetic tree was reconstructed by MrBayes.

Cyanobacterium HN held the highest 16S rRNA gene similarity with *Lyngbya* sp. CENA128T with the value of 99%, revealing that cyanobacterium HN might belong to *Lyngbya* sp. The phylogenetic trees based on the 16S rRNA gene sequences, reconstructed with the Bayesian MCMC methods, showed that cyanobacterium HN fell into the clade comprising *Lyngbya* species and formed a stable clade with *Lyngbya* sp. CENA128T (Figure S1.1B). According to these results, cyanobacterium HN belonged to *Lyngbya* sp.

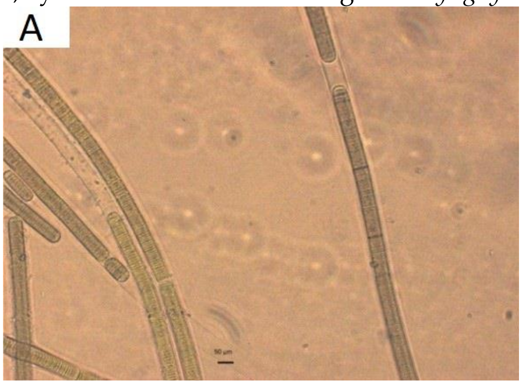

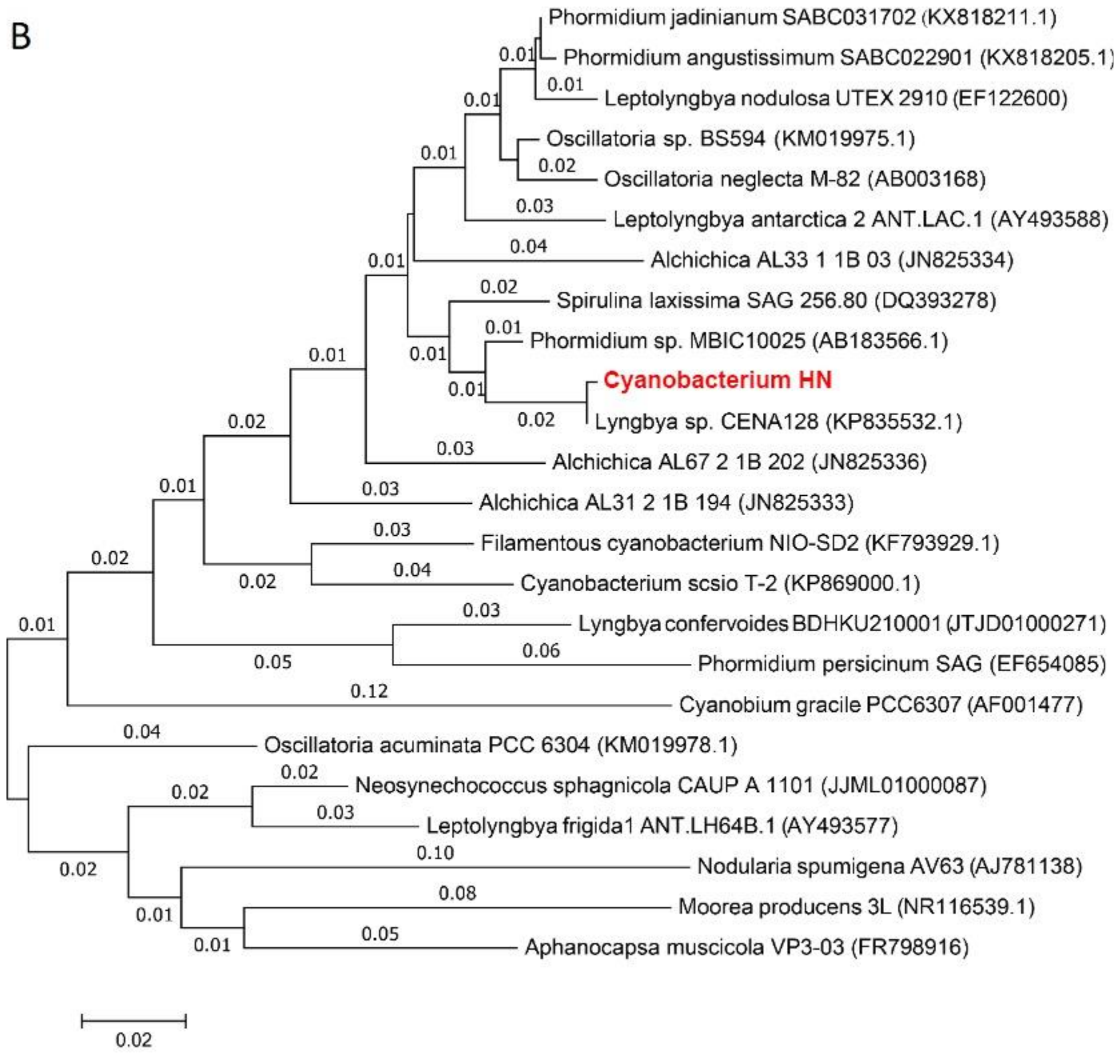

Figure S1.1. (A) *Lyngbya* sp. collected in Harbor of Hainan. The specie, as identified based on morphological features, is shown in light micrographs. (B) Bayesian phylogenetic tree of *Lyngbya* sp. HN and its close relatives. Bootstrap was set as 20,000 replicates. Bar, 0.02 substitutions per nucleotide position.

1.2. Extraction and Isolation

Cyanobacterium freeze-dried powder (150 g) was extracted with CH_2Cl_2 /MeOH (1:1, *v* /v). The obtained extract was dissolved in 1 L of MeOH/H_2O (9:1, v /v) and extracted with CH_2Cl_2 (3 × 1 L) to gain the CH_2Cl_2 extract (20 g) which was subjected to vacuum liquid chromatography (VLC) over silica gel to obtain seven subfractions (F. A-G). The the separation conditions of the gradient used gradients of PE/EtOAc (5:1, 2:1, 1:1, 1:2, 1:5, 0:1, *v* /v).F.B (2,000mg) was further separated by reversed-phase octadecylsilyl silica (ODS) (UV detection at 254 nm, flow rate 20 mL/min , 10%-100% MeCN/H_2O ,180 min) to acquire 17 subfractions (F.B.1-17). Subsequently, the subfraction F.B.7 (231mg) was purified by semi-preparative HPLC (Shimadzu silgreen C-18, 70% MeCN/H_2O, 2.0 mL/min, UV detection at 190 nm) to collect oscillatoxin J (8 mg), oscillatoxin K (15.2 mg), oscillatoxin L (3.7 mg), oscillatoxin M (25.4 mg).

1.3. Bioassays

1.3.1. Ion Channel Experiment

1.3.1.1. Cell culture

For culturing mouse L cells lacking thymidine kinase (Ltk^-) cells stably expressing human Kv1.5 channels (Ltk^-/Kv1.5), 10% fetal bovine serum (FBS), 10,000 U/ml penicillin G and 10 mg/ml streptomycin were added into Dulbecco's Modified Eagle Media (DMEM). Ltk^-/Kv1.5 cells were cultured in a 37 °C humidified incubator set to 5% CO_2. 100 μg/ml Genetic (G418) was added into the culture media to select transgenic cells. When the cells grew to 75% - 85% confluent, they were passaged for subsequent electrophysiological records.μ1.3.1.2. Electrophysiology

1.3.1.2. Electrophysiology

Ltk− cells cultured for at least 24 h could be used for currents recording. For Kv1.5 potassium currents recording, the recording micropipettes were pulled with a P97 microelectrode puller (Sutter, CA, USA) with a resistance about 3 MΩ when filling with internal solution containing: KCl 140 mM, $MgCl_2$ 1 mM, EGTA 5 mM, HEPES 10 mM, MgATP 1 mM (pH was adjusted to 7.25 by KOH). The bath solution contained: NaCl 137 mM, KCl 5.4 mM, $CaCl_2$ 1.8 mM, $MgCl_2$ 1 mM, Glucose 10 mM, HEPES 10 mM (pH was adjusted to 7.4 by NaOH). Kv1.5 currents were recorded at room temperature (22–24°C) by PulseMaster (Version 2.65, Heka, Lambrecht, Germany) via an EPC-10 USB amplifier (Heka, Lambrecht, Germany). To reduce recording errors, cells with seal resistance above 1 GΩ and series resistance was fully compensated above 80% were used. Leak compensation was used to compensate the leak current and to subtract the capacitive artifacts

1.3.1.3. Data analysis

Cells with amplitudes above 1 nA, series resistance less than 10 MΩ and leak compensation below 200 pA were chosen for data analysis. Kv1.5 currents were evoked by a 300 ms depolarizing pulse from -50 mV to 50 mv in 20 mV increments from a holding potential of -70 mV. The current amplitude at the end of 300-ms pulse was measured. The IC_{50} and Hill coefficient (*n*) was obtained by fitting concentration dependence data to the equation of I (%) = $1/\{1+(IC_{50}/[D])^{n}\}$ in which I (%) is the percent inhibition of current (I (%) = $[1-I_{drug}/I_{control}] \times 100$) at test potential and [D] represents various drug concentrations. The measured error at the end of relative short 300 ms pulse was limited and the fitted IC_{50} was acceptable. For steady-state activation curves were fitted with the Boltzmann function: $G/G_{max}=1/\{1+\exp[-(V-V1/2)/k]\}$, where V is a membrane potential and the Nernst K^+ equilibrium potential EK was calculated as−80 mV. G was determined by the following equation: G=I/(V-EK). Figures were drawn by Origin (Version 7.5, OriginLab Co., MA, USA). All values were indicated as mean ± SEM and a value of $P < 0.05$ was considered to be significant.

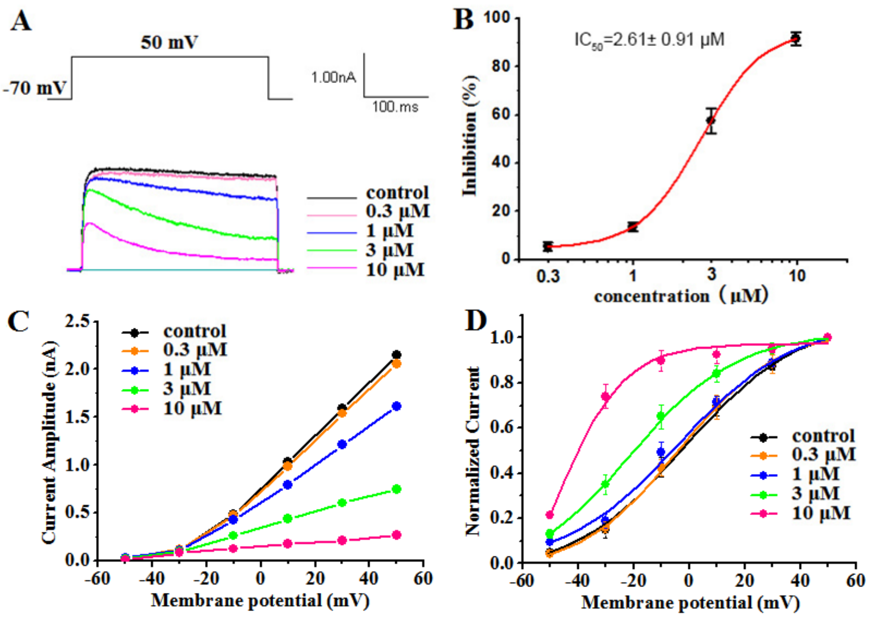

Figure S1.3.1. (A) Kv1.5 currents were evoked by a 300 ms depolarizing pulse from -50 mV to 50 mv in 20 mV increments from a holding potential of -70 mV in the absence and presence of 0.3 μM, 1 μM, 3 μM, and 10 μM oscillatoxin J (1). The current amplitudes were measured at the end of the 300-ms pulse at 50 mV. (B) Concentration-Inhibition curve expressed in %. The abscissa represents the concentration, and the ordinate represents the percentage of Kv1.5 current that is blocked at different concentrations of oscillatoxin J (1). (C) Voltage dependency of oscillatoxin J (1) on Kv 1.5 currents. (D) Normalized activation curves obtained by plotting the current at the end of 300 ms pulse relative to its maximal value against the pulse voltages in control and oscillatoxin J (1) treatments. Data points represent mean ± SEM of 3 to 5 measurements, and the inhibitory effect showed an IC_{50} value of 2.61 ± 0.91 μM.

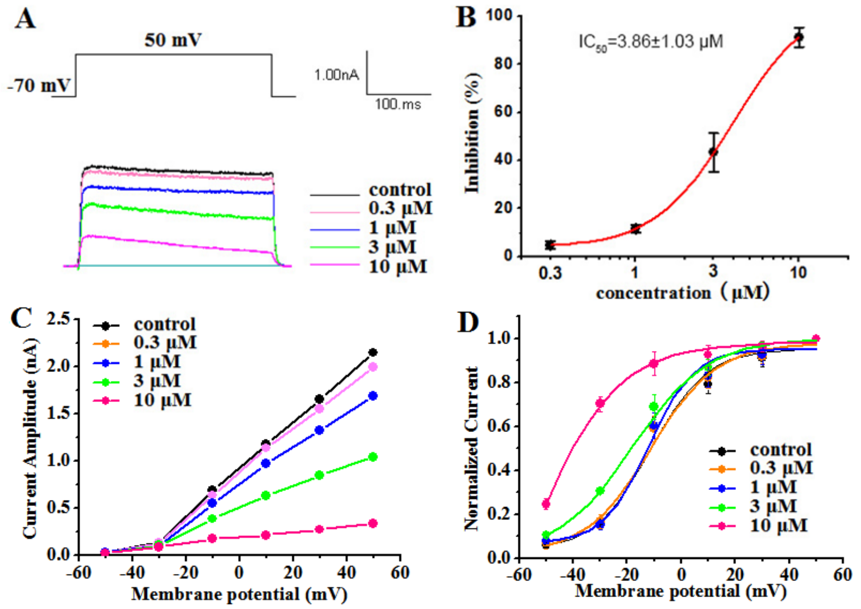

Figure S1.3.2. (A) Kv1.5 currents were evoked by a 300 ms depolarizing pulse from -50 mV to 50 mv in 20 mV increments from a holding potential of -70 mV in the absence and presence of 0.3 μM, 1 μM, 3 μM, and 10 μM oscillatoxin K (2). The current amplitudes were measured at the end of the 300-ms pulse at 50 mV. (B) Concentration-Inhibition curve expressed in %. The abscissa represents the concentration, and the ordinate represents the percentage of Kv1.5 current that is blocked at different concentrations of oscillatoxin K (2). (C) Voltage dependency

of oscillatoxin K (2) on Kv 1.5 currents. (D) Normalized activation curves obtained by plotting the current at the end of 300 ms pulse relative to its maximal value against the pulse voltages in control and oscillatoxin K (2) treatments. Data points represent mean ± SEM of 3 to 5 measurements, and the inhibitory effect showed an IC_{50} value of 3.86 ± 1.03 μM.

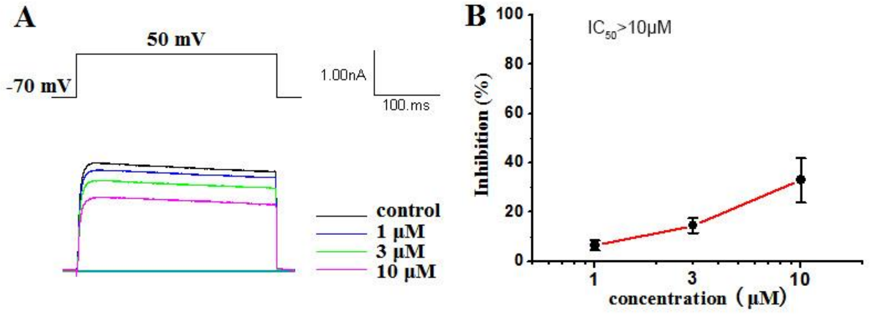

Figure S1.3.3. Kv1.5 currents were evoked by a 300 ms depolarizing pulse from -50 mV to 50 mv in 20 mV increments from a holding potential of -70 mV in the absence and presence of 0.3 μM, 1 μM, 3 μM, and 10 μM oscillatoxin L (**3**). The current amplitudes were measured at the end of the 300-ms pulse at 50 mV. (**B**) Concentration-Inhibition curve expressed in %. The abscissa represents the concentration, and the ordinate represents the percentage of Kv1.5 current that is blocked at different concentrations of oscillatoxin L (**3**). Data points represent mean ± SEM of 3 to 5 measurements, and the inhibitory effect showed IC_{50} > 10 μM.

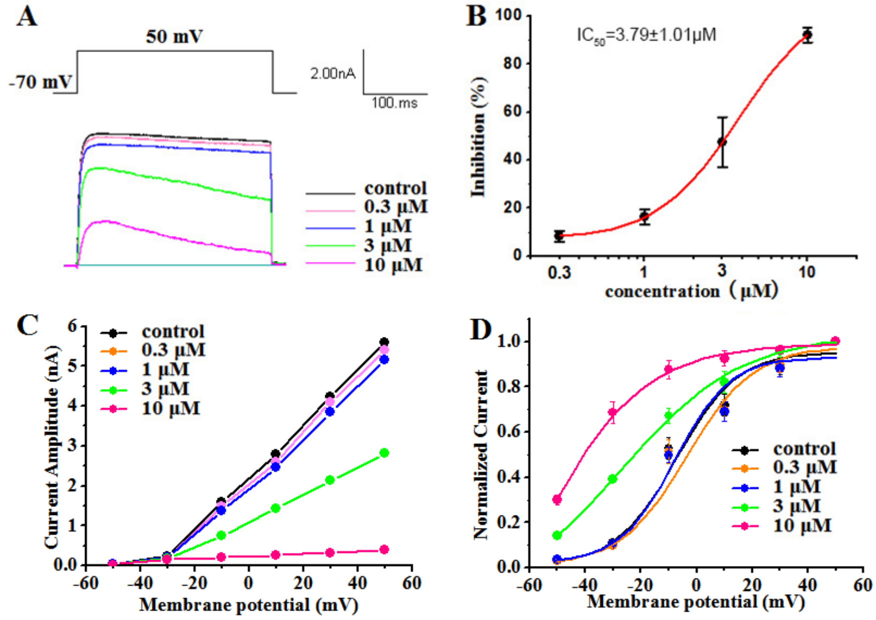

Figure S1.3.4. (A)Kv1.5 currents were evoked by a 300 ms depolarizing pulse from -50 mV to 50 mv in 20 mV increments from a holding potential of -70 mV in the absence and presence of 0.3 μM, 1 μM, 3 μM, and 10 μM oscillatoxin M (4). The current amplitudes were measured at the end of the 300-ms pulse at 50 mV. (B) Concentration-Inhibition curve expressed in %. The abscissa represents the concentration, and the ordinate represents the percentage of Kv1.5 current that is blocked at different concentrations of oscillatoxin M (4). (C) Voltage dependency of oscillatoxin M (4) on Kv 1.5 currents. (D) Normalized activation curves obtained by plotting the current at the end of 300 ms pulse relative to its maximal value against the pulse voltages in control and oscillatoxin M (4) treatments. Data points represent mean ± SEM of 3 to 5 measurements, and the inhibitory effect showed an IC_{50} value of 3.79 ± 1.01 μM.

1.3.2. Brine Shrimp Cytotoxicity Assay

Commercially available *Artemia salina* (*A. salina)* or brine shrimp cysts were purchased and cultivated in 3.2% of saline water. Before cultivation, the saline was aerated, and then cysts were kept at room temperature for 24 h. For toxicity screening, hatched larvae were collected and introduced in saline water. Add saline water and equivalent larvae in per well of 96 wells to make test culture plate. Aplysiatoxins with 0.1 μM, 1 μM, 10 μM, 30 μM was added to test culture plate, while DMSO and dichloromethane were added as blank control test and positive control separately. After 24 h in 25 °C, the percent of survival of *A. salina* was calculated.

Table S1.3.2.1. Effect of compounds **1-4** to *Artemia salina (A. salina). A. salina* were treated with indicated concentration (0.1 μM, 1 μM, 10 μM, 30 μM) of dichloromethane (DCM), debromoaplysiatoxin (DAT), oscillatoxin J, oscillatoxin K, oscillatoxin L, oscillatoxin M, Methylenechloride, Control for 24 h. The percentage of A. *salina* with all different kinds of aplysiatoxins (ATXs).

Compounds	Percentage of Survival of *Artemia salina* after 24 h (μM)			
	**0.1**	**1**	**10**	**30**
Debromoaplysiatoxin	87.19±0.74	2.96±2.1	0	0
oscillatoxin J	96.62±0.37	95.87±0.7	95±1.52	93.61±5.11
oscillatoxin K	96.63±0.05	96.05±0.07	95.23±0.19	89.97±7.12
oscillatoxin L	97.74±1.60	98.89±1.57	96.37±0.28	73.38±9.64
oscillatoxin M	95.13±1.09	96.29±0.11	96.15±0.12	82.27±6.13
Methylenechloride	0	0	0	0
Control	98.92 ±1.92			

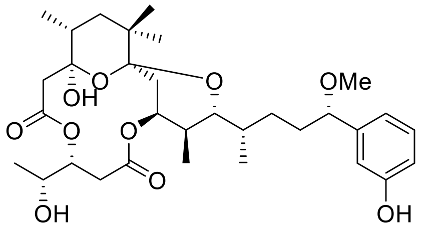

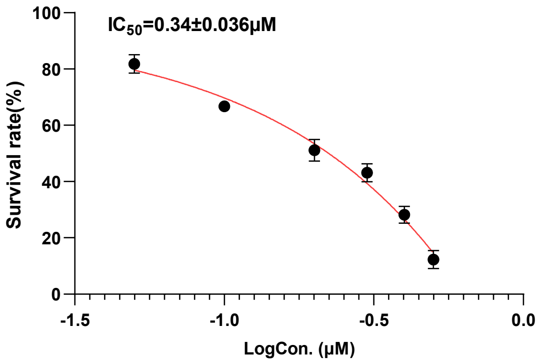

Figure S1.3.2.1. Effect of debromoaplysiatoxin to *Artemia salina (A. salina). A. salina* were treated with debromoaplysiatoxin with indicated concentration 0.05 μM, 0.1 μM, 0.2 μM, 0.3 μM, 0.4 μM, 0.5 μM and inhibitory effect showed IC_{50} value of 0.034 ± 0.036 μM.

1.4. Methods for NMR Calculation of oscillatoxin K-M (2-4)

The calculations were performed by using the density functional theory (DFT) as carried out in the Gaussian 09 (Gaussian, Wallingford, CT, USA). Conformational searches were run by employing the "systematic" procedure implemented in Spartan'14 (Spartan Software, San Francisco, CA, USA) using MMFF. All MMFF minima were reoptimized with DFT calculations at the B3LYP/6-31G (d) level. The stable conformations obtained at the B3LYP/6-31G (d) level were further used in magnetic shielding constants at the B3LYP/6-311++G (2d, p) level.

Table S1.4.1.DFT-optimized structures and thermodynamic parameters for low-energy conformers of **2a**

Conformers	Conf.2a-1	Conf.2a-2
DFT-optimized structures	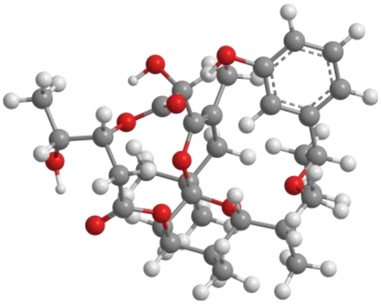	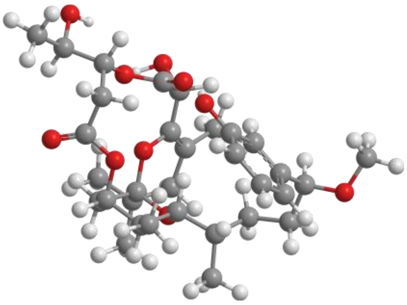
Population	93.5%	6.5%
Total energy (a.u.)	-1999.35086378	-1999.34834249
Sum of electronic and zero-point energies (a.u.)	-1998.594724	-1998.592745
Sum of electronic and thermal energies (a.u.)	-1998.552579	-1998.550328
Sum of electronic and thermal enthalpies (a.u.)	-1998.551635	-1998.549383
Sum of electronic and thermal free energies (a.u.)	-1998.667503	-1998.665821

Table S1.4.2. DFT-optimized structures and thermodynamic parameters for low-energy conformers of **2b**

Conformers	Conf.2b-1	Conf.2b-2
DFT-optimized structures	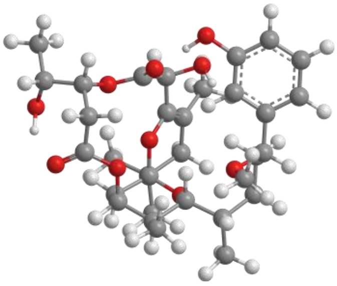	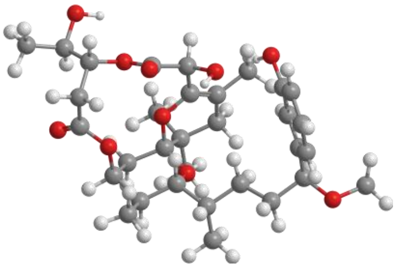
Population	94.0%	6.0%
Total energy (a.u.)	-1999.35261912	-1999.35003158
Sum of electronic and zero-point energies (a.u.)	-1998.596277	-1998.594558
Sum of electronic and thermal energies (a.u.)	-1998.554437	-1998.552223

Sum of electronic and thermal enthalpies (a.u.)	-1998.553493	-1998.551279
Sum of electronic and thermal free energies (a.u.)	-1998.667558	-1998.667846

Table S1.4.3. The calculated 13C NMR data for 2.

no.	δexp	δcal		δscal		corrected error		t distribution		probability	
		2a	2b	2a	2b	2a	2b	2a	2b	2a	2b
1	171.06	185.20	188.07	174.00	176.12	2.94	5.06	0.89	0.97	0.11	0.03
2	70.17	77.87	77.90	72.26	72.31	2.09	2.14	0.81	0.81	0.19	0.19
3	140.15	151.82	150.31	142.35	140.53	2.20	0.38	0.82	0.56	0.18	0.44
4	110.28	119.25	119.28	111.48	111.29	1.20	1.01	0.69	0.67	0.31	0.33
5	39.90	41.82	42.25	38.09	38.71	-1.81	-1.19	0.78	0.69	0.22	0.31
6	35.73	41.91	42.70	38.17	39.14	2.44	3.41	0.84	0.92	0.16	0.08
7	99.88	107.75	107.86	100.58	100.54	0.70	0.66	0.62	0.61	0.38	0.39
8	30.40	33.32	34.64	30.04	31.54	-0.36	1.14	0.56	0.68	0.44	0.32
9	74.34	80.48	80.26	74.73	74.53	0.39	0.19	0.57	0.53	0.43	0.47
10	34.13	39.52	38.18	35.91	34.87	1.78	0.74	0.77	0.62	0.23	0.38
11	72.01	76.08	73.49	70.57	68.15	-1.44	-3.86	0.73	0.94	0.27	0.06
12	33.52	37.24	36.45	33.75	33.24	0.23	-0.28	0.54	0.55	0.46	0.45
13	30.30	38.24	37.19	34.70	33.94	4.40	3.64	0.96	0.93	0.04	0.07
14	35.97	40.58	40.67	36.91	37.22	0.94	1.25	0.65	0.70	0.35	0.30
15	85.00	92.91	93.02	86.52	86.55	1.52	1.55	0.74	0.74	0.26	0.26
16	144.39	153.12	154.04	143.59	144.05	-0.80	-0.34	0.63	0.56	0.37	0.44
17	118.47	122.06	121.48	114.15	113.37	-4.32	-5.10	0.96	0.98	0.04	0.02
18	129.58	134.75	136.52	126.18	127.55	-3.40	-2.03	0.92	0.80	0.08	0.20
19	114.54	120.39	120.98	112.56	112.90	-1.98	-1.64	0.80	0.75	0.20	0.25
20	156.16	167.69	169.18	157.40	158.31	1.24	2.15	0.70	0.81	0.30	0.19
21	114.37	116.00	111.98	108.41	104.42	-5.96	-9.95	0.99	1.00	0.01	0.00
22	11.98	16.08	15.27	13.70	13.29	1.72	1.31	0.76	0.71	0.24	0.29
23	13.37	14.59	14.48	12.28	12.54	-1.09	-0.83	0.68	0.64	0.32	0.36
24	22.76	22.56	23.19	19.84	20.75	-2.92	-2.01	0.88	0.80	0.12	0.20
25	24.40	26.03	26.36	23.13	23.74	-1.27	-0.66	0.70	0.61	0.30	0.39
26	16.85	19.84	18.68	17.25	16.50	0.40	-0.35	0.57	0.56	0.43	0.44
27	169.84	183.63	184.21	172.51	172.48	2.67	2.64	0.86	0.86	0.14	0.14
28	36.89	41.60	41.65	37.88	38.14	0.99	1.25	0.66	0.70	0.34	0.30
29	74.02	80.71	82.50	74.95	76.63	0.93	2.61	0.65	0.86	0.35	0.14
30	68.93	76.08	76.10	70.57	70.60	1.64	1.67	0.75	0.76	0.25	0.24
31	19.93	21.67	21.54	18.99	19.19	-0.94	-0.74	0.65	0.62	0.35	0.38
32	56.73	57.24	57.24	52.70	52.83	-4.03	-3.90	0.95	0.94	0.05	0.06
		Product of probabilities								2.90×10^{-23}	4.09×10^{-25}
		Bayes's theorem probability (%)								98.61	1.39

Table S1.4.4. DFT-optimized structures and thermodynamic parameters for low-energy conformers of **3a**.

Conformers	Conf. 3a-1	Conf. 3a-2
DFT-optimized structures	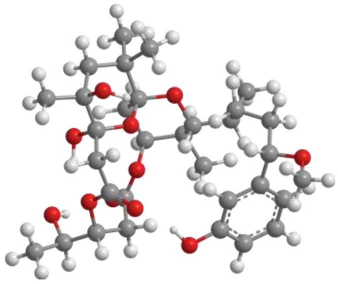	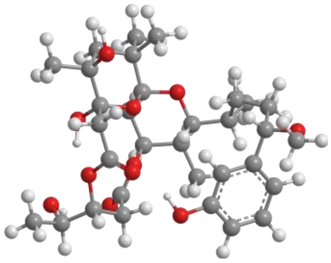
Population	82.1%	17.9%
Total energy (a.u.)	-2075.79550136	-2075.79406721
Sum of electronic and zero-point energies (a.u.)	-2075.010696	-2075.009816
Sum of electronic and thermal energies (a.u.)	-2074.968139	-2074.967164
Sum of electronic and thermal enthalpies (a.u.)	-2074.967195	-2074.966220
Sum of electronic and thermal free energies (a.u.)	-2075.082454	-2075.081278

Table S1.4.5. DFT-optimized structures and thermodynamic parameters for low-energy conformers of **3b.**

Conformers	Conf. 3b-1	Conf. 3b-2
DFT-optimized structures	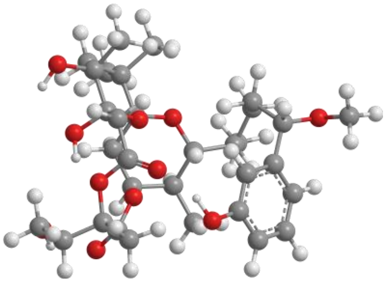	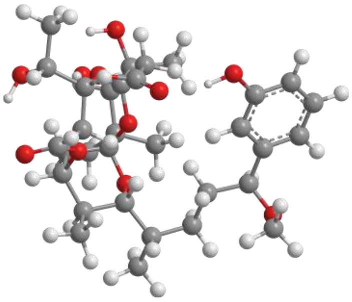
Population	93.5%	6.5%
Total energy (a.u.)	-2075.79539386	-2075.79287556
Sum of electronic and zero-point energies (a.u.)	-2075.009303	-2075.007115
Sum of electronic and thermal energies (a.u.)	-2074.967250	-2074.964836
Sum of electronic and thermal enthalpies (a.u.)	-2074.966305	-2074.963892

Sum of electronic and thermal free energies (a.u.)	-2075.079910	-2075.079091

Table S1.4.6. DFT-optimized structures and thermodynamic parameters for low-energy conformers of **3c**.

Conformers	Conf. 3c
DFT-optimized structures	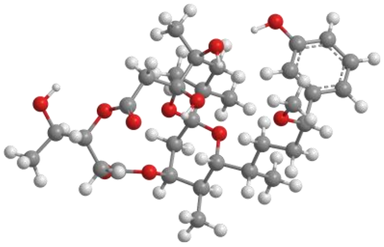
Population	100%
Total energy (a.u.)	-2075.79746716
Sum of electronic and zero-point energies (a.u.)	-2075.012771
Sum of electronic and thermal energies (a.u.)	-2074.970138
Sum of electronic and thermal enthalpies (a.u.)	-2074.969194
Sum of electronic and thermal free energies (a.u.)	-2075.084564

Table S1.4.7. DFT-optimized structures and thermodynamic parameters for low-energy conformers of **3d**.

Conformers	Conf. 3d-1	Conf. 3d-2	Conf. 3d-3
DFT-optimized structures	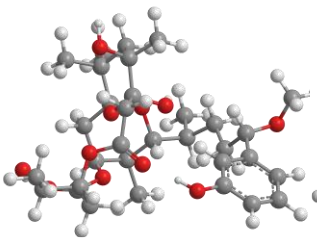	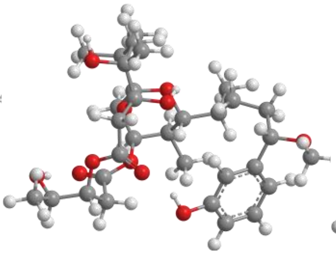	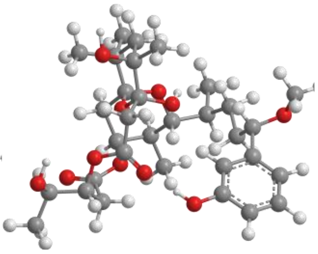
Population	50.2%	39.8%	10.0%
Total energy (a.u.)	-2075.79653565	-2075.79631663	-2075.79501727
Sum of electronic and zero-point energies (a.u.)	-2075.012476	-2075.011662	-2075.011380

Sum of electronic and thermal energies (a.u.)	-2074.969458	-2074.968995	-2074.968097
Sum of electronic and thermal enthalpies (a.u.)	-2074.968514	-2074.968051	-2074.967153
Sum of electronic and thermal free energies (a.u.)	-2075.084978	-2075.082667	-2075.084476

Table S1.4.8. The calculated ^{13}C NMR data for **3.**

no.	δexp	δcal				δscal				corrected error				t distribution				probability			
		3a	3b	3c	3d	3a	3b	3c	3d	3a	3b	3c	3d	3a	3b	3c	3d	3a	3b	3c	3d
1	174.20	181.70	182.09	184.47	183.03	172.14	173.24	175.70	174.47	-2.06	-0.96	1.50	0.27	0.80	0.66	0.74	0.55	0.20	0.34	0.26	0.45
2	40.00	44.17	44.17	45.82	43.35	39.81	40.43	42.48	38.77	-0.19	0.43	2.48	-1.23	0.53	0.57	0.85	0.70	0.47	0.43	0.15	0.30
3	101.10	110.03	108.56	105.66	109.92	103.18	102.44	99.98	103.45	2.08	1.34	-1.12	2.35	0.81	0.71	0.68	0.83	0.19	0.29	0.32	0.17
4	72.61	78.50	75.58	77.71	80.57	72.84	70.69	73.12	74.93	0.23	-1.92	0.51	2.32	0.54	0.79	0.59	0.83	0.46	0.21	0.41	0.17
5	43.40	48.72	52.21	48.70	48.92	44.19	48.18	45.24	44.19	0.79	4.78	1.84	0.79	0.63	0.97	0.78	0.63	0.37	0.03	0.22	0.37
6	37.70	46.92	47.72	45.41	46.35	42.45	43.85	42.08	41.69	4.75	6.15	4.38	3.99	0.97	0.99	0.96	0.94	0.03	0.01	0.04	0.06
7	102.00	109.44	109.54	110.09	111.45	102.61	103.38	104.24	104.93	0.61	1.38	2.24	2.93	0.60	0.72	0.82	0.89	0.40	0.28	0.18	0.11
8	32.77	37.87	37.55	34.31	36.21	33.75	34.06	31.42	31.84	0.98	1.29	-1.35	-0.93	0.66	0.71	0.72	0.65	0.34	0.29	0.28	0.35
9	73.30	82.60	82.66	79.30	79.04	76.79	77.50	74.65	73.45	3.49	4.20	1.35	0.15	0.92	0.95	0.71	0.53	0.08	0.05	0.29	0.47
10	33.90	40.88	40.56	39.18	42.02	36.64	36.96	36.09	37.49	2.74	3.06	2.19	3.59	0.87	0.89	0.82	0.93	0.13	0.11	0.18	0.07
11	72.60	78.64	73.89	79.30	75.73	72.98	69.06	74.65	70.23	0.38	-3.54	2.05	-2.37	0.56	0.92	0.80	0.84	0.44	0.08	0.20	0.16
12	33.50	37.87	34.91	41.54	39.91	33.75	31.52	38.36	35.44	0.25	-1.98	4.86	1.94	0.54	0.80	0.97	0.79	0.46	0.20	0.03	0.21
13	30.00	34.00	29.89	31.51	35.65	30.02	26.69	28.73	31.30	0.02	-3.31	-1.27	1.30	0.50	0.91	0.70	0.71	0.50	0.09	0.30	0.29
14	36.10	38.57	36.90	38.63	42.84	34.42	33.43	35.57	38.29	-1.68	-2.67	-0.53	2.19	0.76	0.86	0.59	0.82	0.24	0.14	0.41	0.18
15	84.80	92.79	88.13	89.56	90.18	86.59	82.77	84.50	84.27	1.79	-2.03	-0.30	-0.53	0.77	0.80	0.55	0.59	0.23	0.20	0.45	0.41
16	144.10	153.76	153.60	150.20	151.46	145.26	145.81	142.77	143.80	1.16	1.71	-1.33	-0.30	0.69	0.76	0.71	0.55	0.31	0.24	0.29	0.45
17	118.21	121.11	119.94	122.15	117.30	113.85	113.40	115.82	110.61	-4.36	-4.81	-2.39	-7.60	0.96	0.97	0.84	1.00	0.04	0.03	0.16	0.00
18	129.90	136.25	134.96	134.96	134.18	128.41	127.86	128.13	127.01	-1.49	-2.04	-1.77	-2.89	0.73	0.80	0.77	0.88	0.27	0.20	0.23	0.12
19	114.70	118.09	118.08	119.36	118.64	110.94	111.61	113.14	111.91	-3.76	-3.09	-1.56	-2.79	0.93	0.90	0.74	0.87	0.07	0.10	0.26	0.13
20	155.97	165.25	165.57	165.70	167.32	156.32	157.33	157.67	159.21	0.35	1.36	1.70	3.24	0.56	0.72	0.76	0.91	0.44	0.28	0.24	0.09
21	114.77	120.61	119.06	116.12	117.82	113.36	112.55	110.03	111.12	-1.41	-2.22	-4.74	-3.65	0.72	0.82	0.97	0.93	0.28	0.18	0.03	0.07
22	13.80	11.74	15.54	10.19	15.48	8.61	12.87	8.24	11.70	-5.19	-0.93	-5.56	-2.10	0.98	0.65	0.98	0.81	0.02	0.35	0.02	0.19
23	12.20	14.10	14.00	14.70	16.04	10.88	11.39	12.57	12.24	-1.32	-0.81	0.37	0.04	0.71	0.63	0.56	0.51	0.29	0.37	0.44	0.49
24	27.10	33.70	32.20	25.06	26.70	29.73	28.91	22.53	22.61	2.63	1.81	-4.57	-4.49	0.86	0.78	0.96	0.96	0.14	0.22	0.04	0.04
25	26.50	30.39	28.08	28.16	28.77	26.55	24.94	25.50	24.62	0.05	-1.56	-1.00	-1.88	0.51	0.74	0.66	0.78	0.49	0.26	0.34	0.22
26	18.10	24.29	23.67	28.16	30.24	20.69	20.70	25.50	26.04	2.59	2.60	7.40	7.94	0.86	0.86	1.00	1.00	0.14	0.14	0.00	0.00
27	169.90	182.73	182.82	178.00	183.20	173.13	173.95	169.48	174.63	3.23	4.05	-0.42	4.73	0.91	0.95	0.57	0.97	0.09	0.05	0.43	0.03
28	35.40	42.93	43.09	37.77	42.02	38.62	39.40	34.75	37.49	3.22	4.00	-0.65	2.09	0.91	0.94	0.61	0.81	0.09	0.06	0.39	0.19

29	74.60	80.33	80.26	88.92	79.70	74.60	75.19	83.89	74.09	0.00	0.59	9.29	-0.51	0.50	0.60	1.00	0.59	0.50	0.40	0.00	0.41
30	67.90	76.88	76.32	71.55	76.47	71.28	71.40	67.20	70.95	3.38	3.50	-0.70	3.05	0.91	0.92	0.62	0.89	0.09	0.08	0.38	0.11
31	26.40	21.29	21.31	18.09	21.68	17.80	18.42	15.83	17.73	-8.60	-7.98	-10.57	-8.67	1.00	1.00	1.00	1.00	0.00	0.00	0.00	0.00
32	56.90	57.01	58.78	58.52	58.90	52.16	54.50	54.67	53.88	-4.74	-2.40	-2.23	-3.02	0.97	0.84	0.82	0.89	0.03	0.16	0.18	0.11
Product of probabilities																		2.69×10^{-26}	1.45×10^{-29}	1.46×10^{-30}	1.32×10^{-30}
Bayes's theorem probability (%)																		1.00	0.54×10^{-3}	5.41×10^{-5}	4.91×10^{-5}

Table S1.4.9. DFT-optimized structures and thermodynamic parameters for low-energy conformers of **4a**.

Conformers	Conf. 4a-1	Conf. 4a-2
DFT-optimized structures	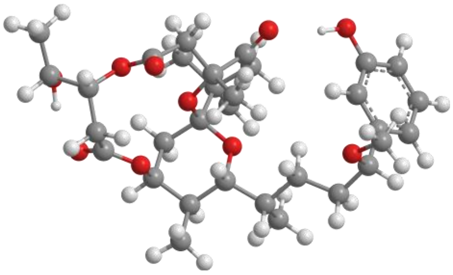	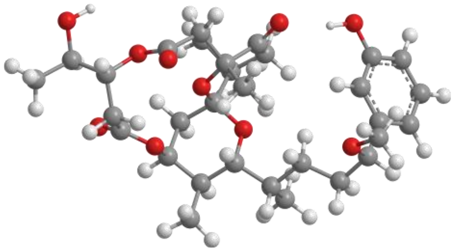
Population	88.6%	11.4%
Total energy (a.u.)	-1999.37151132	-1999.36957664
Sum of electronic and zero-point energies (a.u.)	-1998.615334	-1998.613811
Sum of electronic and thermal energies (a.u.)	-1998.573674	-1998.571890
Sum of electronic and thermal enthalpies (a.u.)	-1998.572730	-1998.570945
Sum of electronic and thermal free energies (a.u.)	-1998.686961	-1998.685687

Table S1.4.10. DFT-optimized structures and thermodynamic parameters for low-energy conformers of **4b**.

Conformers	Conf.4b-1	Conf. 4b-2	Conf. 4b-3
DFT-optimized structures	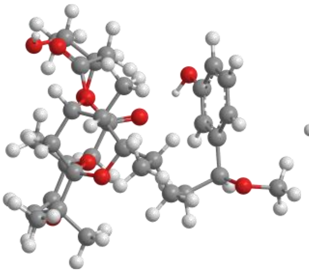	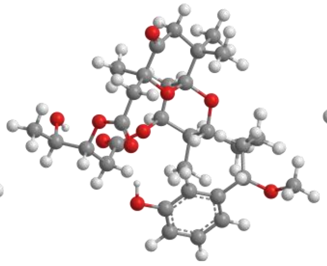	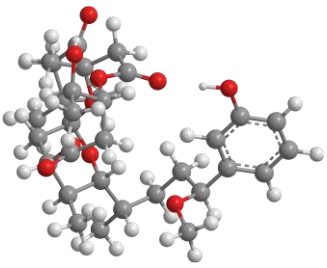

Population	75.7%	15.4%	18.9%
Total energy (a.u.)	-1999.35443311	-1999.35293229	-1999.35241143
Sum of electronic and zero-point energies (a.u.)	-1998.597391	-1998.596008	-1998.596379
Sum of electronic and thermal energies (a.u.)	-1998.556145	-1998.554686	-1998.554611
Sum of electronic and thermal enthalpies (a.u.)	-1998.555201	-1998.553741	-1998.553666
Sum of electronic and thermal free energies (a.u.)	-1998.667648	-1998.666747	-1998.668548

Table S1.4.11. The calculated ^{13}C NMR data for **4.**

no.	δexp	δcal		δscal		corrected error		t distribution		probability	
		4a	4b	4a	4b	4a	4b	4a	4b	4a	4b
1	166.16	179.44	183.05	167.16	173.03	1.00	6.87	0.66	0.99	0.34	0.01
2	46.31	47.47	48.88	45.00	45.37	-1.31	-0.94	0.71	0.65	0.29	0.35
3	86.28	88.03	90.17	82.55	84.67	-3.73	-1.61	0.93	0.75	0.07	0.25
4	203.22	232.47	217.54	216.25	205.85	13.03	2.63	1.00	0.86	0.00	0.14
5	44.89	49.69	50.75	47.05	47.15	2.16	2.26	0.82	0.83	0.18	0.17
6	47.11	45.60	56.00	43.26	52.15	-3.85	5.04	0.94	0.97	0.06	0.03
7	108.73	108.02	107.83	101.05	101.46	-7.68	-7.27	1.00	1.00	0.00	0.00
8	31.55	34.35	36.82	32.85	33.90	1.30	2.35	0.71	0.84	0.29	0.16
9	74.16	79.34	81.81	74.50	76.71	0.34	2.55	0.56	0.85	0.44	0.15
10	34.06	40.11	41.16	38.18	38.03	4.12	3.97	0.95	0.94	0.05	0.06
11	73.72	79.45	74.55	74.60	69.80	0.88	-3.92	0.65	0.94	0.35	0.06
12	33.77	39.45	35.08	37.57	32.24	3.80	-1.53	0.94	0.74	0.06	0.26
13	30.03	29.55	29.38	28.41	26.82	-1.62	-3.21	0.75	0.90	0.25	0.10
14	36.23	39.76	36.68	37.86	33.77	1.63	-2.46	0.75	0.85	0.25	0.15
15	84.84	87.68	87.35	82.22	81.98	-2.62	-2.86	0.86	0.88	0.14	0.12
16	144.09	149.55	153.88	139.49	145.28	-4.60	1.19	0.96	0.69	0.04	0.31

17	118.25	122.64	118.83	114.58	111.93	-3.67	-6.32	0.93	0.99	0.07	0.01
18	129.77	135.26	134.48	126.26	126.82	-3.51	-2.95	0.92	0.89	0.08	0.11
19	114.89	122.40	117.66	114.36	110.82	-0.53	-4.07	0.59	0.95	0.41	0.05
20	155.88	166.95	165.47	155.61	156.31	-0.27	0.43	0.55	0.57	0.45	0.43
21	114.58	116.44	120.16	108.84	113.20	-5.74	-1.38	0.98	0.72	0.02	0.28
22	11.28	10.39	16.37	10.66	14.45	-0.62	3.17	0.60	0.90	0.40	0.10
23	13.8	14.35	14.01	14.33	12.20	0.53	-1.60	0.59	0.75	0.41	0.25
24	23.6	26.48	28.99	25.57	26.46	1.97	2.86	0.79	0.88	0.21	0.12
25	25.59	26.43	26.74	25.52	24.31	-0.07	-1.28	0.51	0.70	0.49	0.30
26	27.18	28.45	28.19	27.39	25.69	0.21	-1.49	0.54	0.73	0.46	0.27
27	170.05	184.52	183.05	171.87	173.03	1.82	2.98	0.78	0.89	0.22	0.11
28	35.58	42.06	43.55	39.99	40.30	4.41	4.72	0.96	0.97	0.04	0.03
29	74.04	78.27	79.57	73.51	74.57	-0.53	0.53	0.59	0.59	0.41	0.41
30	68.69	75.65	76.89	71.08	72.02	2.39	3.33	0.84	0.91	0.16	0.09
31	19.1	21.68	21.03	21.12	18.88	2.02	-0.22	0.80	0.54	0.20	0.46
32	56.79	58.81	59.02	55.49	55.02	-1.30	-1.77	0.71	0.77	0.29	0.23
Product of probabilities										3.47×10^{-30}	3.92×10^{-31}
Bayes's theorem probability (%)										0.90	0.10

2. Figures

2.1 Spectra Data of compound ***1.***

Figure S2.1.1 ^{1}H NMR spectrum of compound **1** in $CDCl_3$.

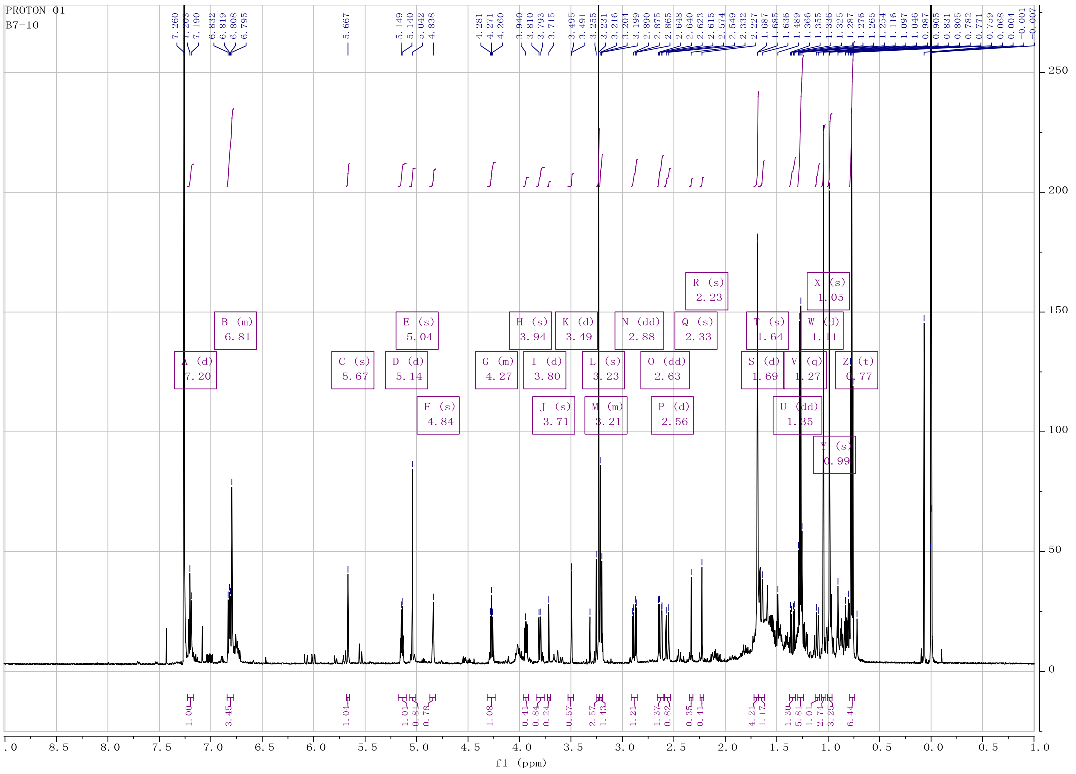

Figure S2.1.2 ^{13}C NMR spectrum of compound **1** in $CDCl_3$.

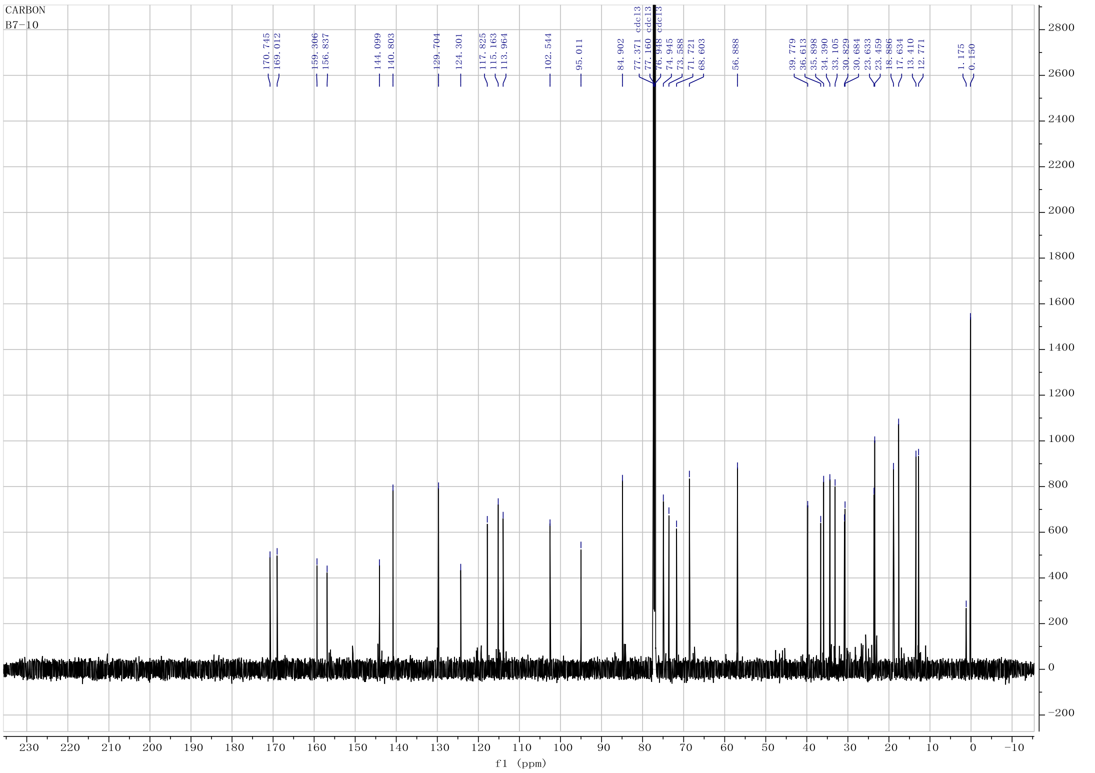

Figure S2.1.3 DEPT spectrum of compound **1** in $CDCl_3$.

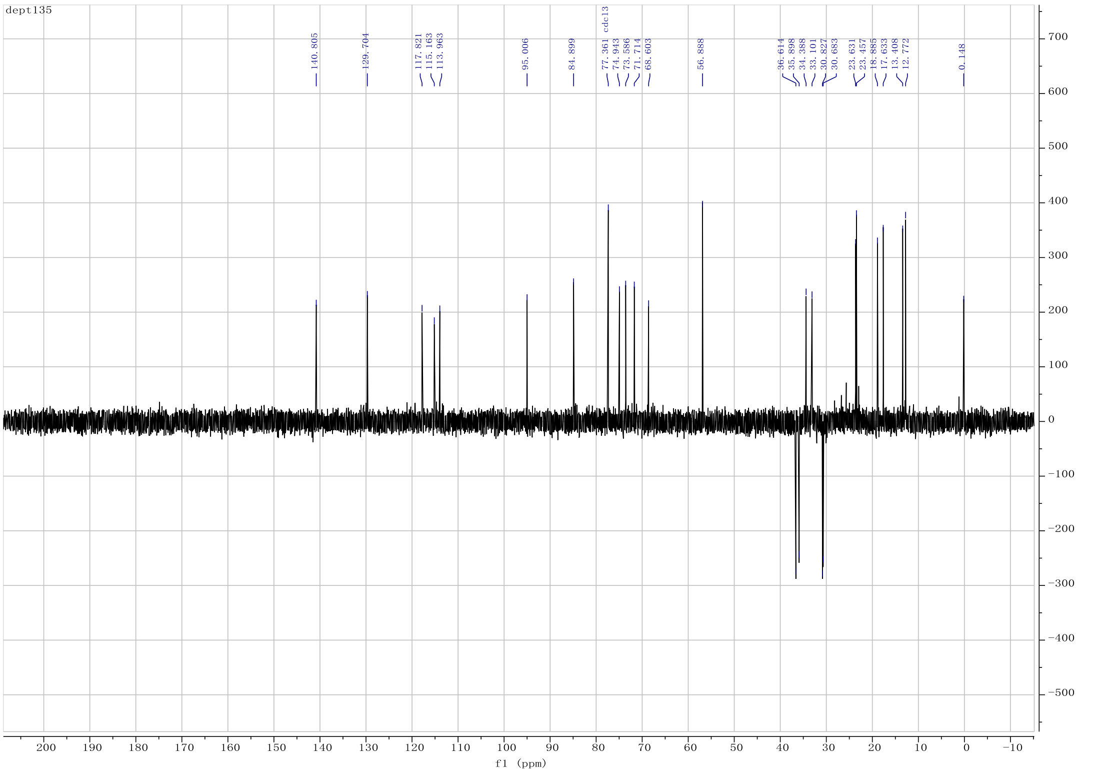

Figure S2.1.4 HSQC spectrum of compound **1** in $CDCl_3$.

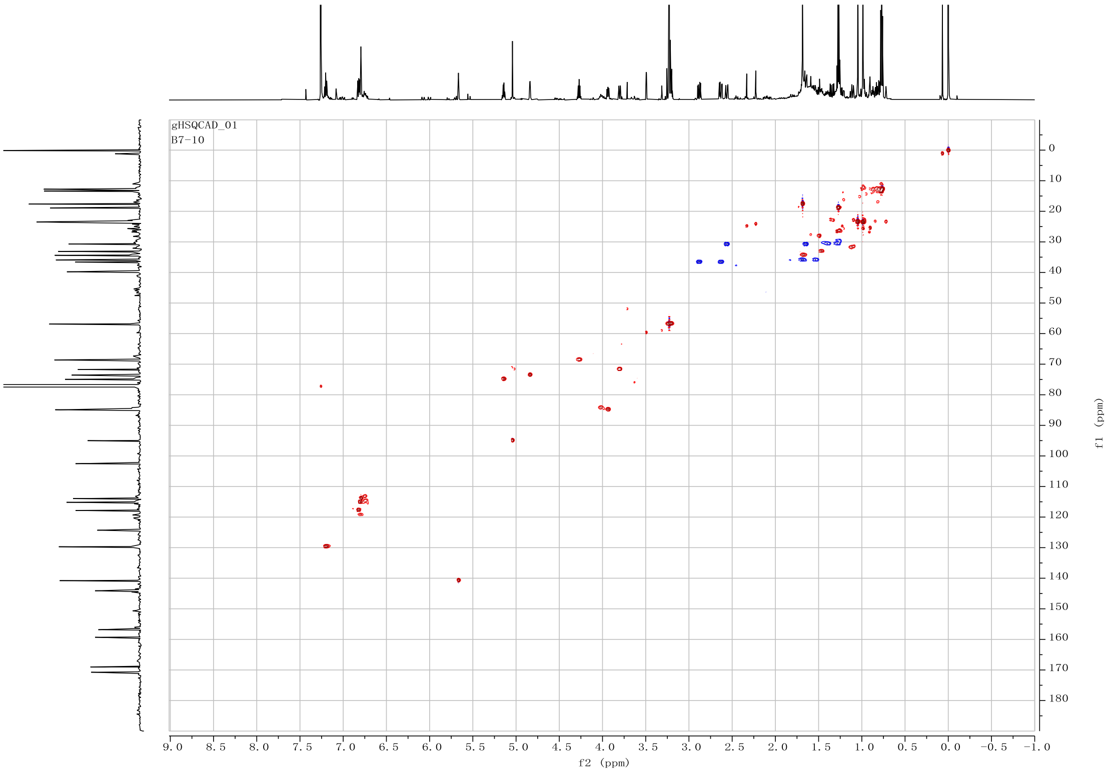

Figure S2.1.5 ^{1}H-^{1}H COSY spectrum of compound **1** in $CDCl_3$.

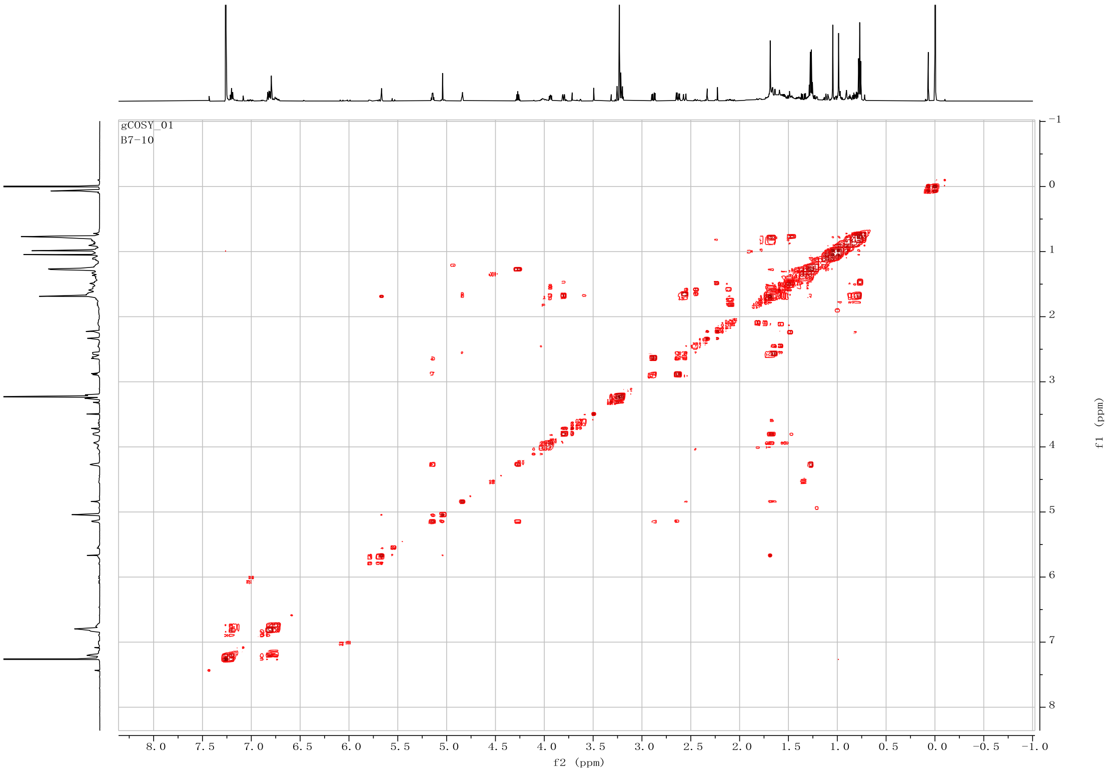

Figure S2.1.6 HMBC spectrum of compound **1** in $CDCl_3$.

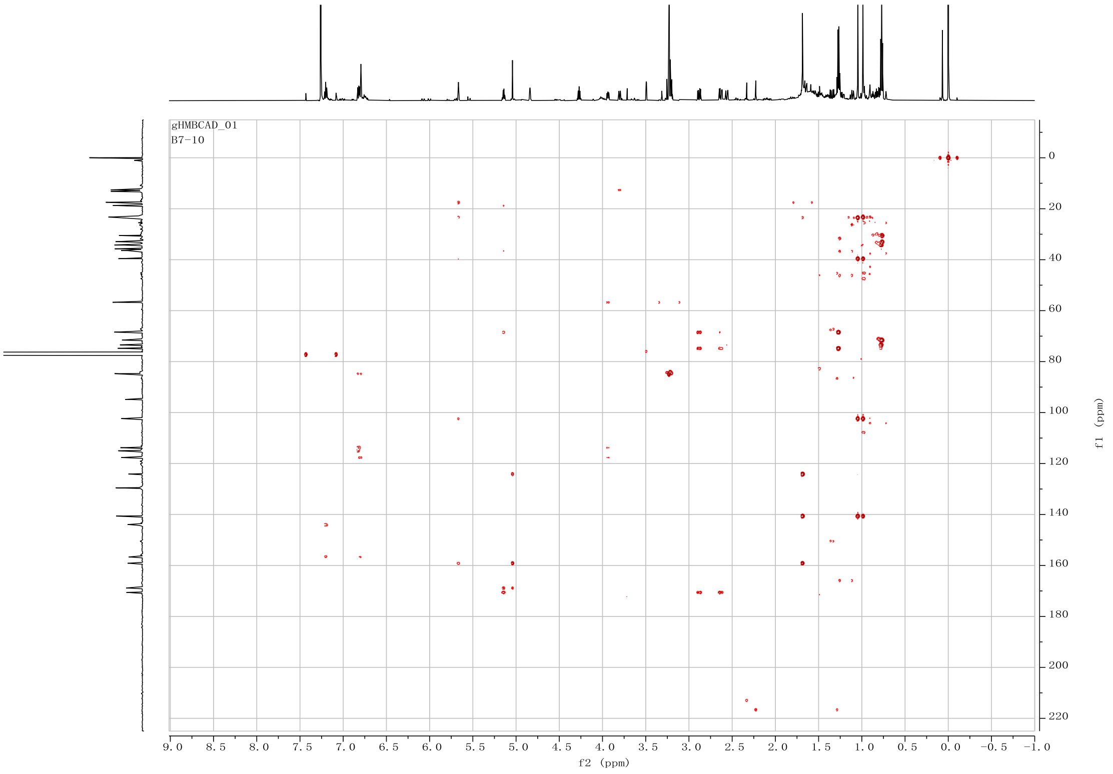

Figure S2.1.7 ROESY spectrum of compound **1** in $CDCl_3$.

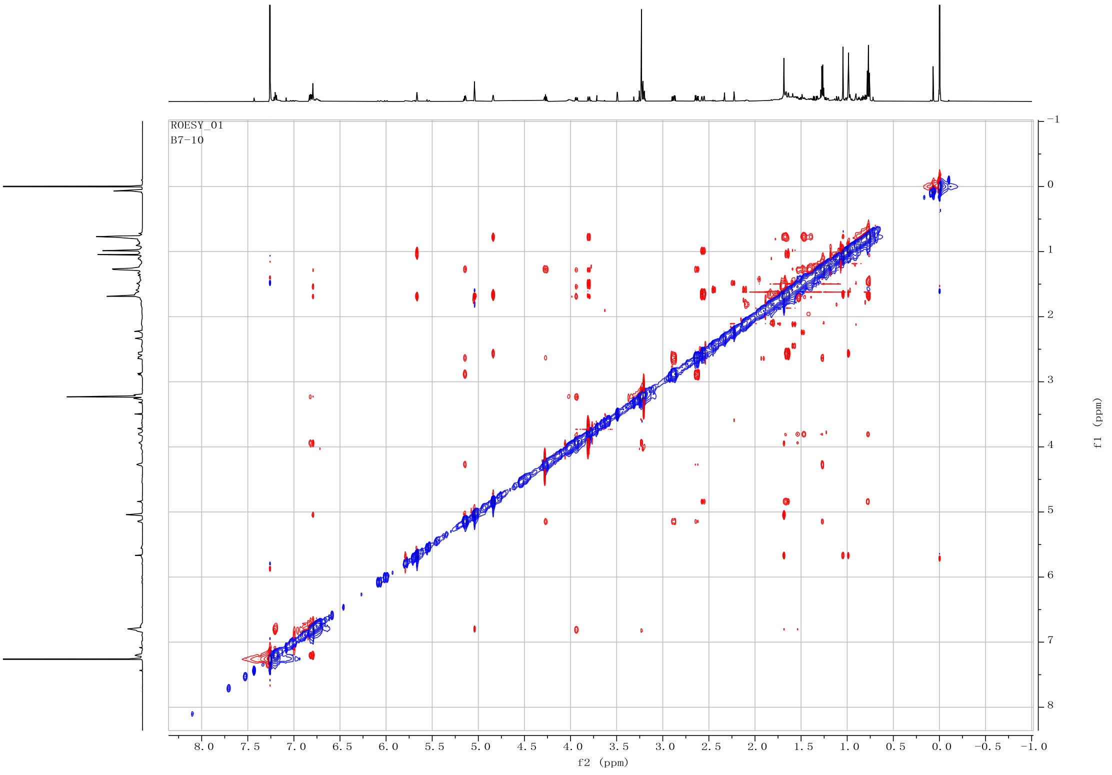

Figure S2.1.8 HRESIMS spectrum of compound **1** in MeOH.

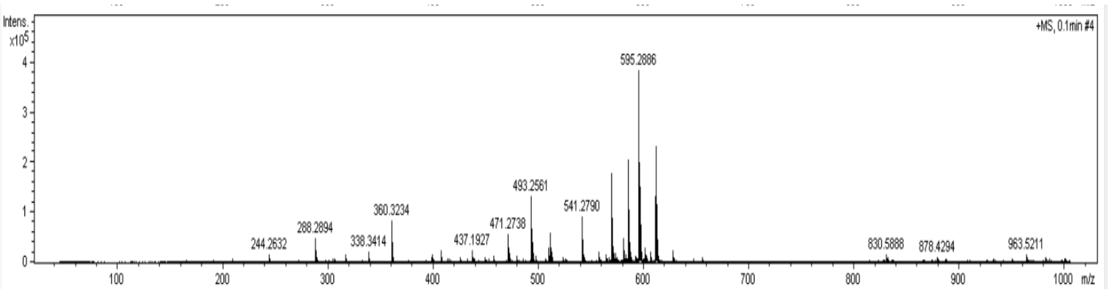

Figure S2.1.9 UV spectrum of **1** in MeOH.

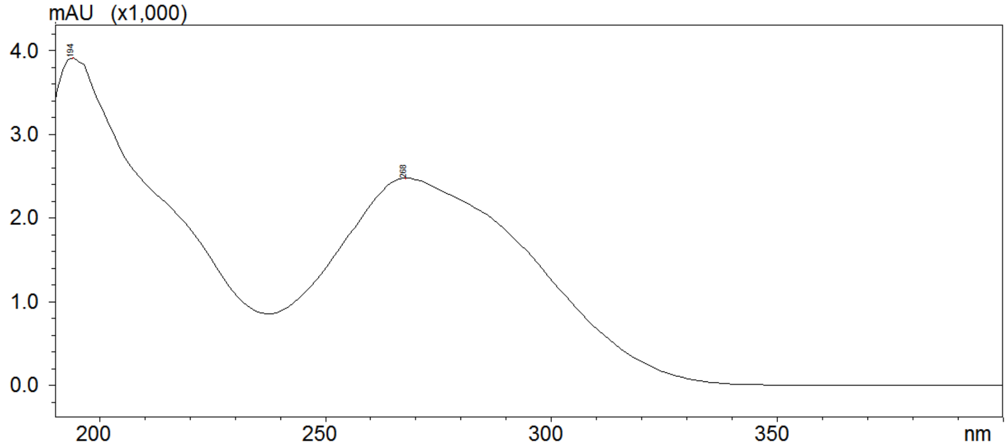

Figure S2.1.10 IR spectrum of **1**.

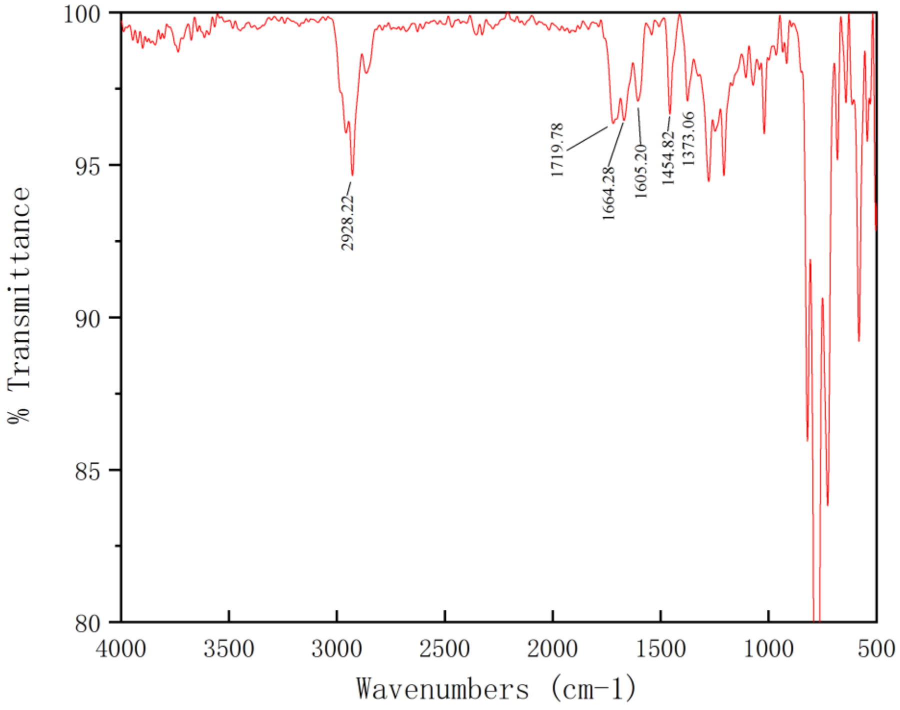

2.2 Spectra Data of compound 2.

Figure S2.2.1 ^{1}H NMR spectrum of compound **2** in $CDCl_3$.

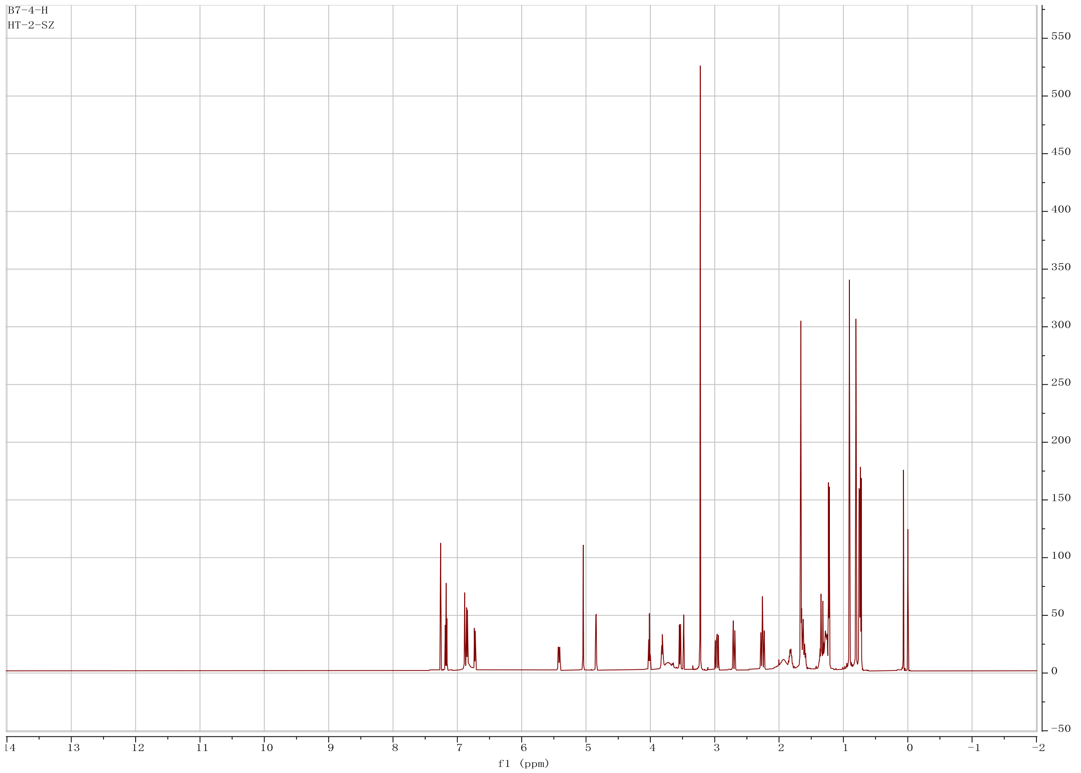

Figure S2.2.2 ^{13}C NMR spectrum of compound **2** in $CDCl_3$.

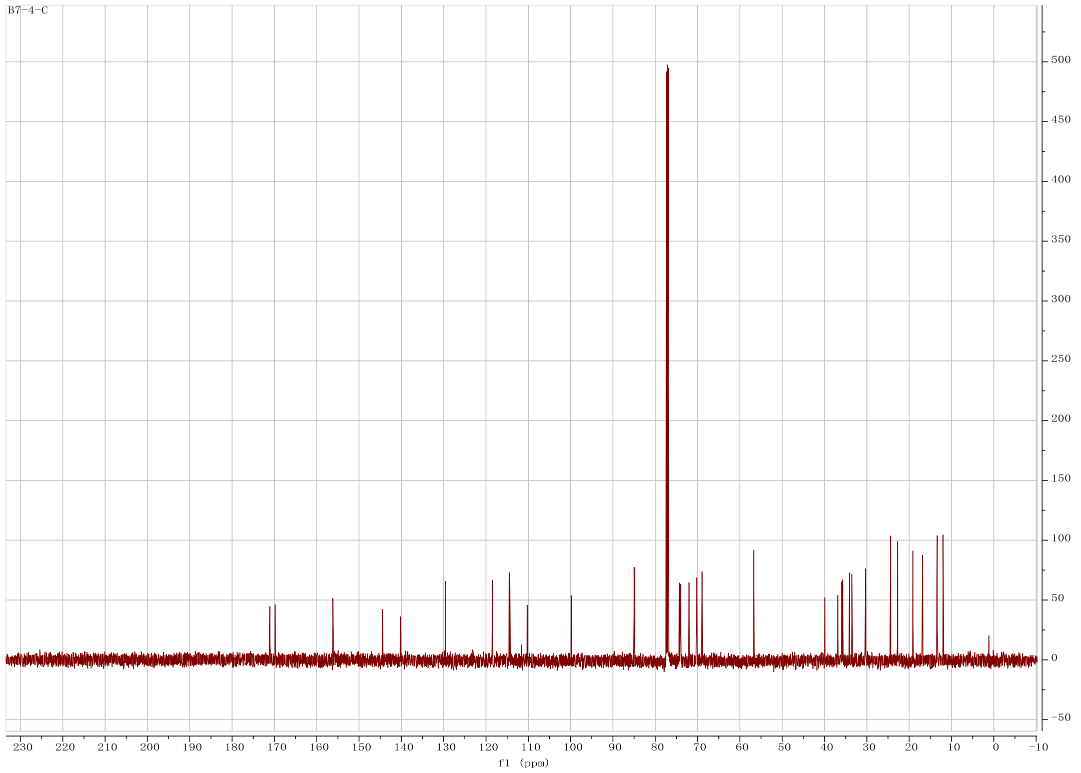

Figure S2.2.3 DEPT spectrum of compound 2 in CDCl3.

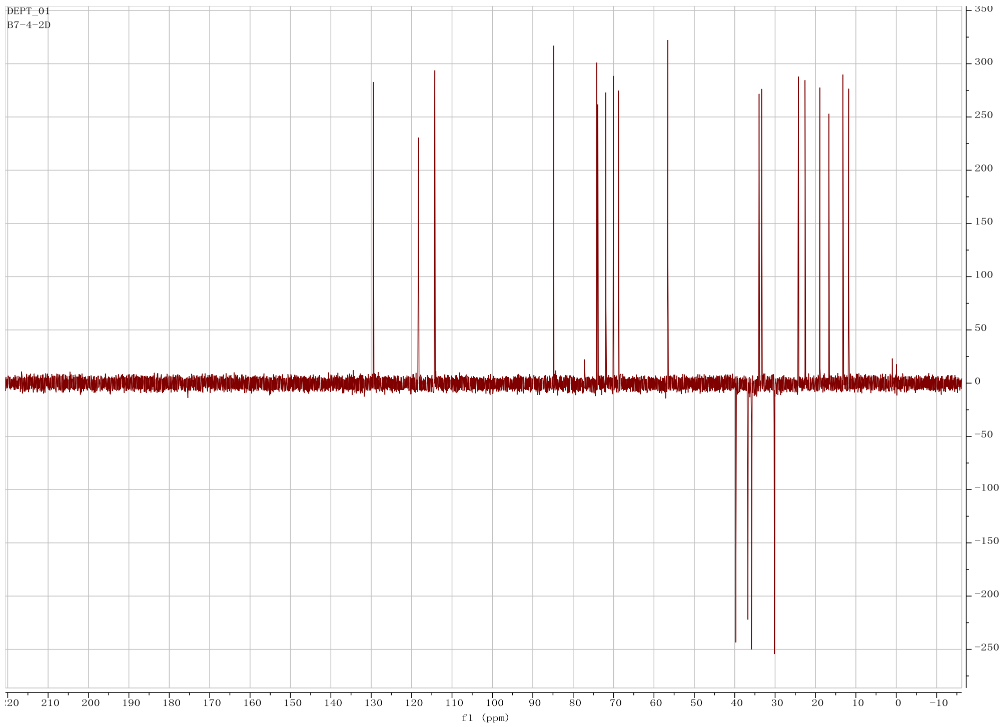

Figure S2.2.4 HSQC spectrum of compound **2** in $CDCl_3$.

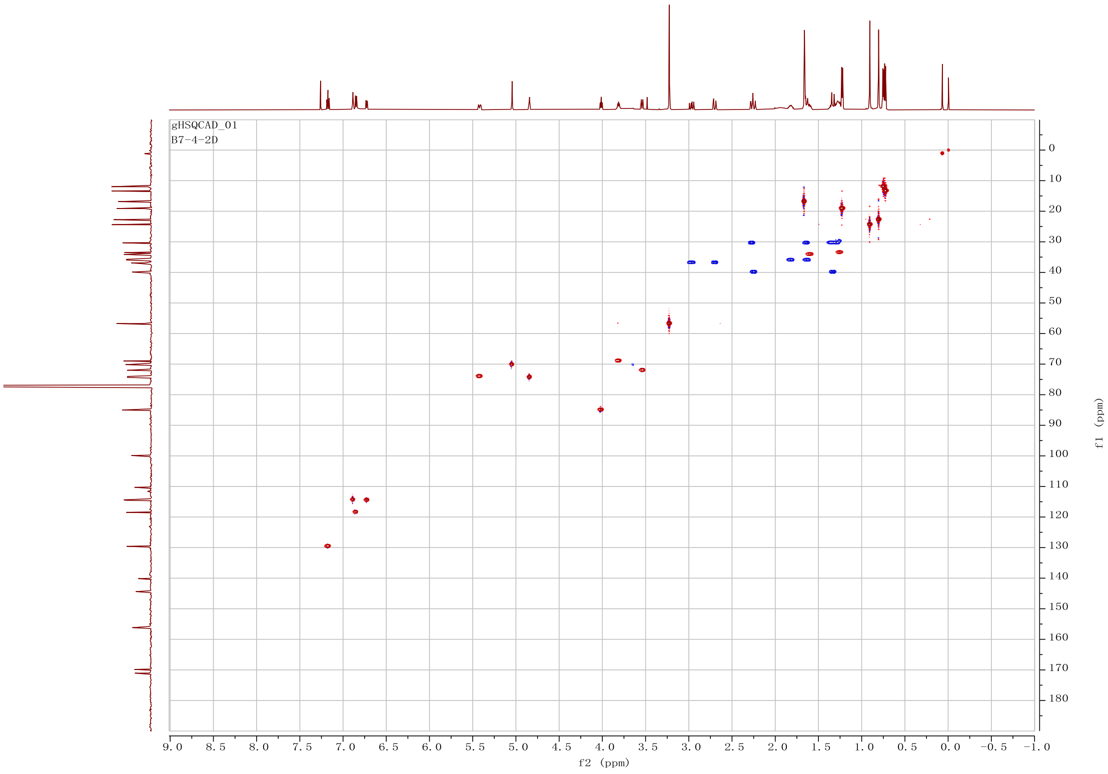

Figure S2.2.5 ^1H-^1H COSY spectrum of compound **2** in $CDCl_3$.

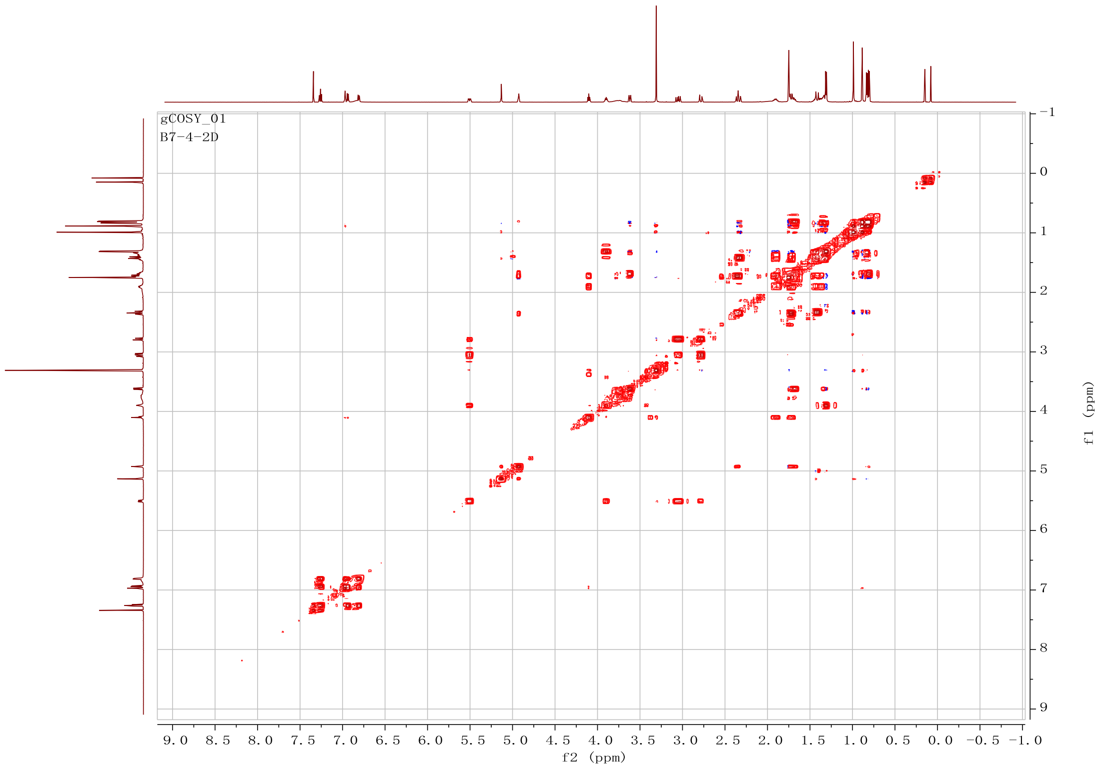

Figure S2.2.6 HMBC spectrum of compound **2** in $CDCl_3$.

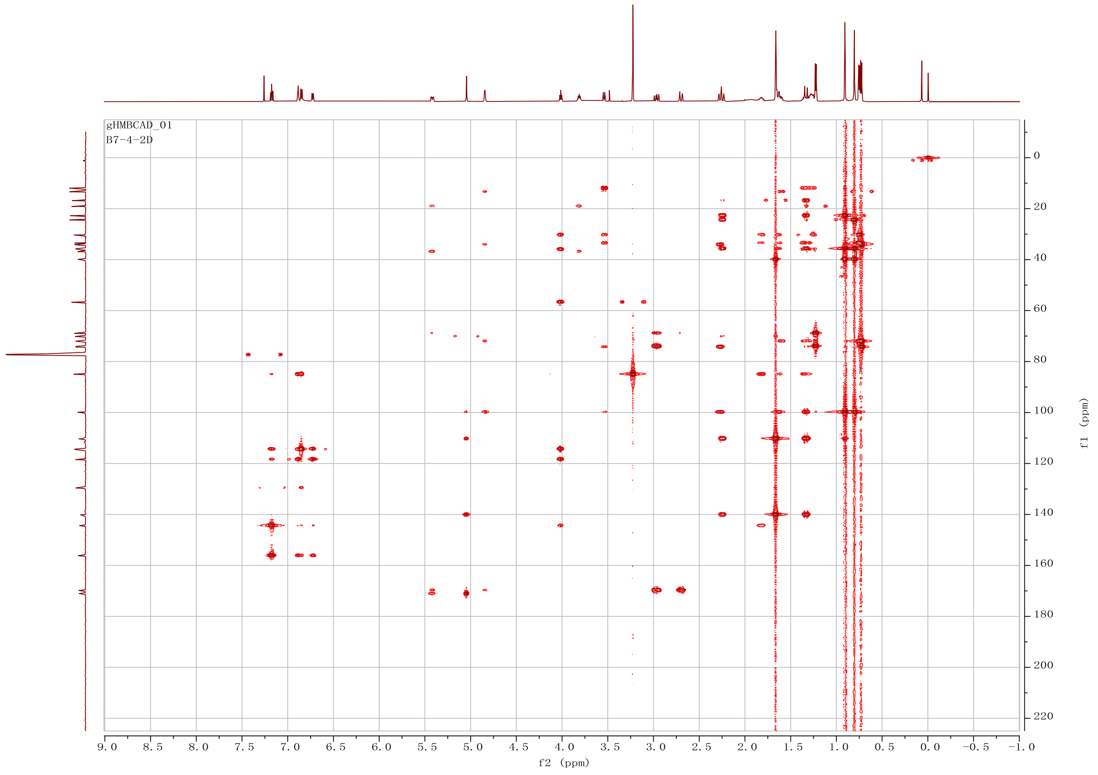

Figure S2.2.7 NOESY spectrum of compound **2** in $CDCl_3$.

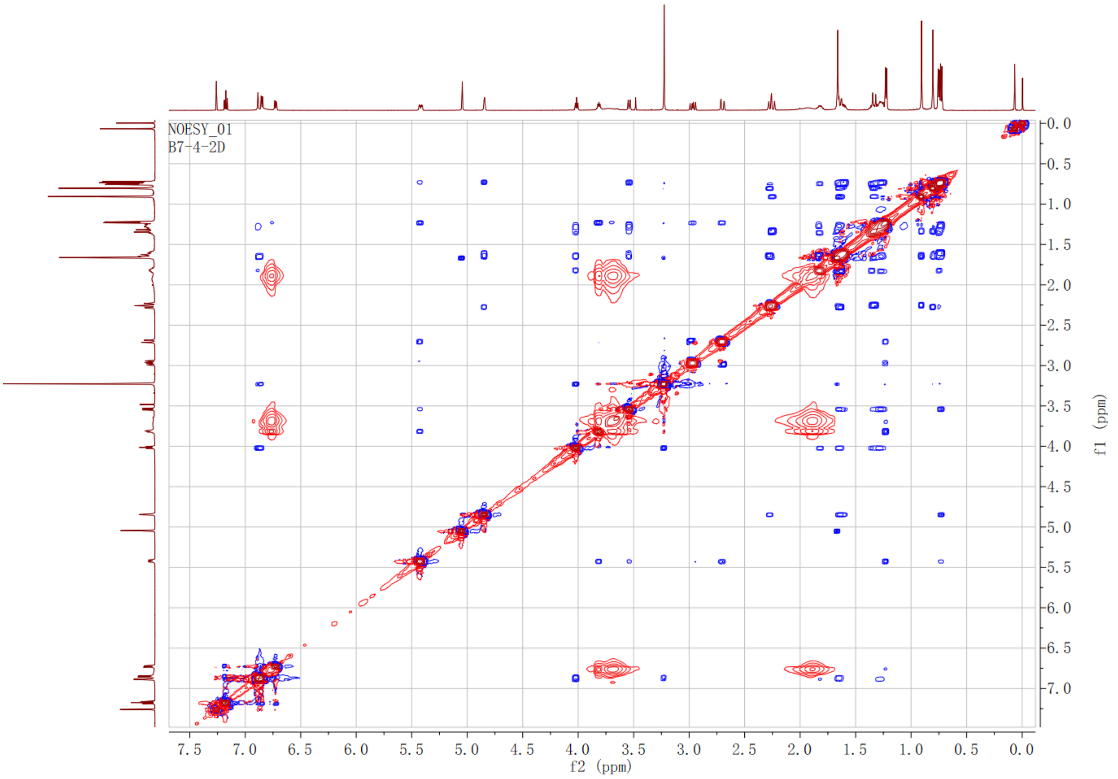

Figure S2.2.8 HRESIMS spectrum of compound **2** in MeOH.

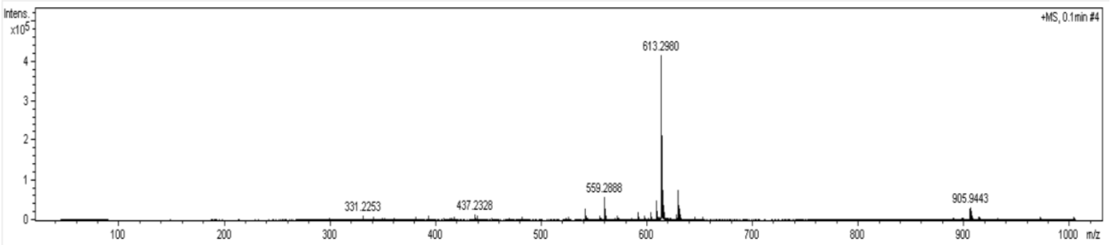

Figure S2.2.9 UV spectrum of **2** in MeOH.

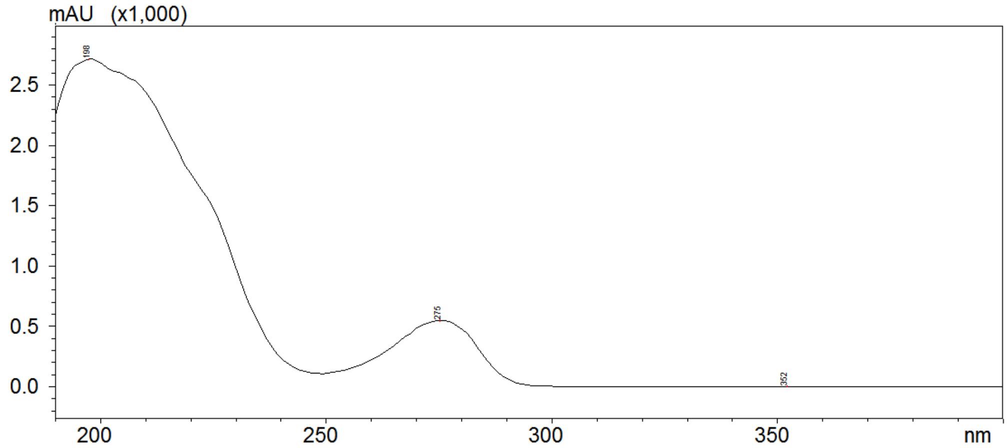

Figure S2.2.10 IR spectrum of **2**.

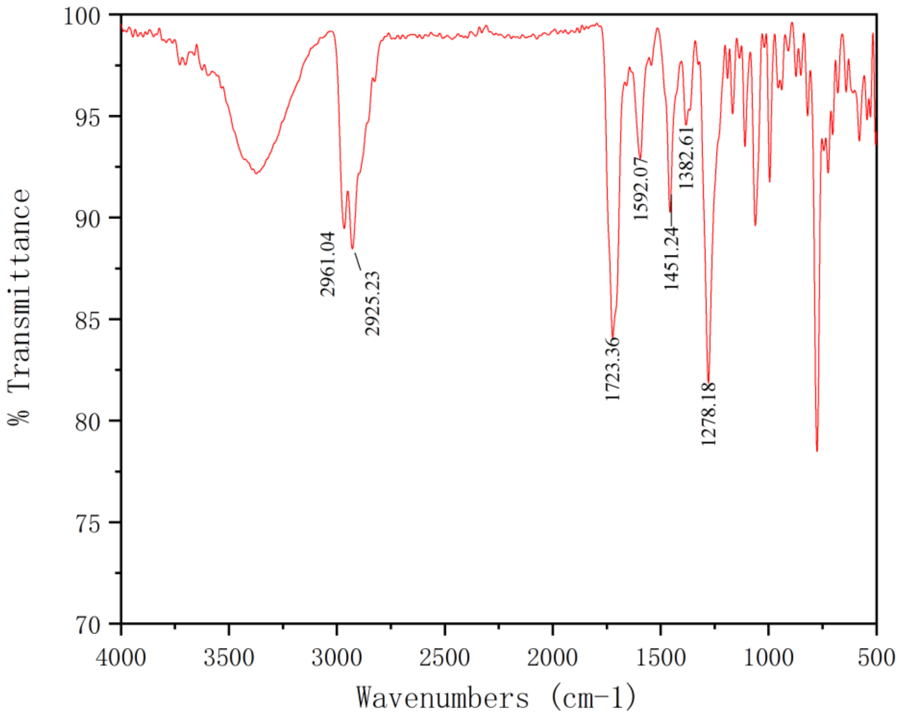

*2.3 Spectra Data of compound **3***

Figure S2.3.1 ^{1}H NMR spectrum of compound **3** in $CDCl_3$.

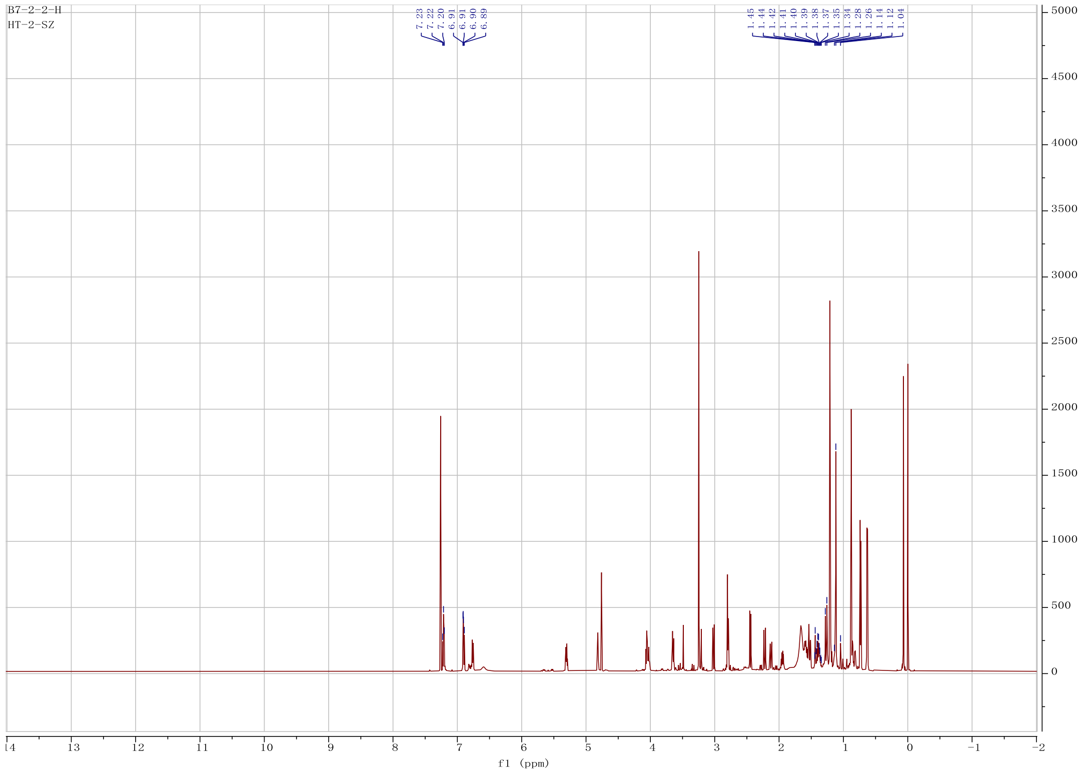

Figure S2.3.2 ^{13}C NMR spectrum of compound **3** in $CDCl_3$.

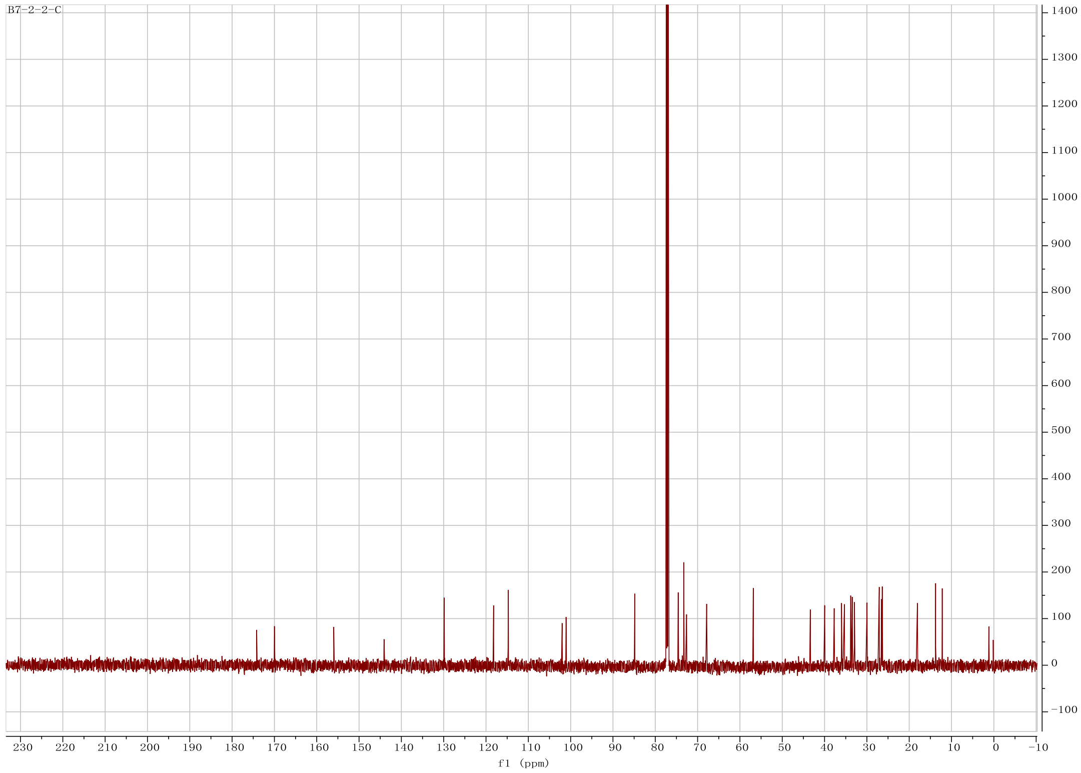

Figure S2.3.3 DEPT spectrum of compound **3** in $CDCl_3$.

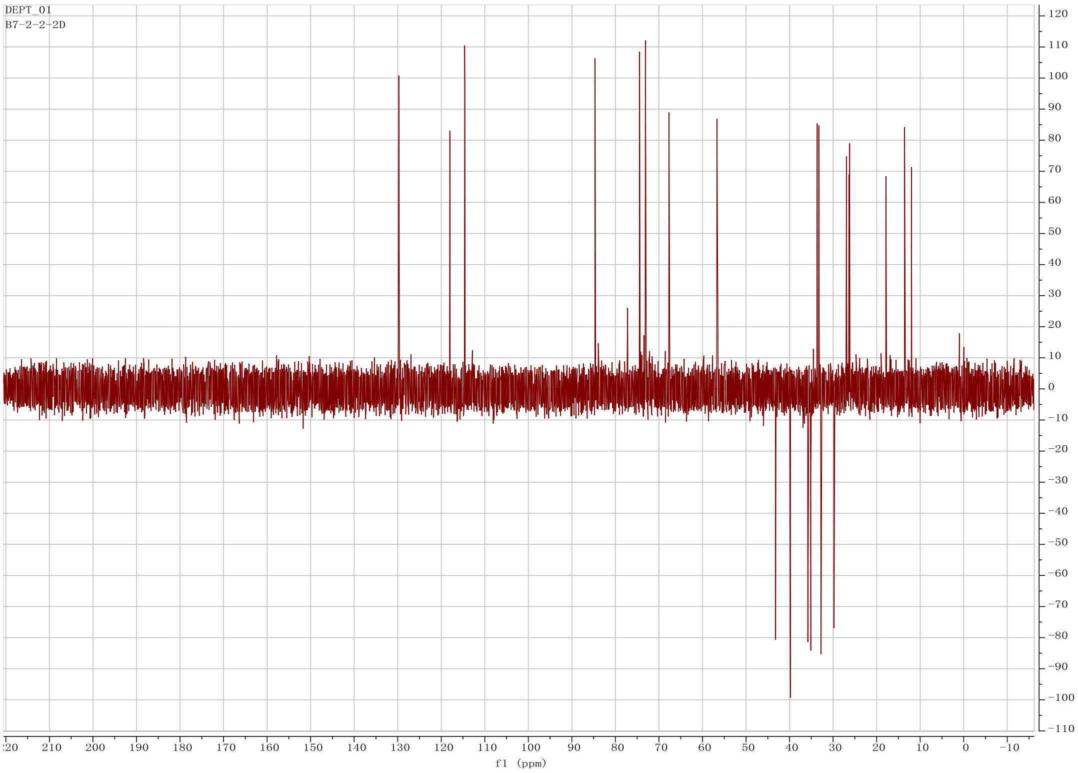

Figure S2.3.4 HSQC spectrum of compound **3** in $CDCl_3$.

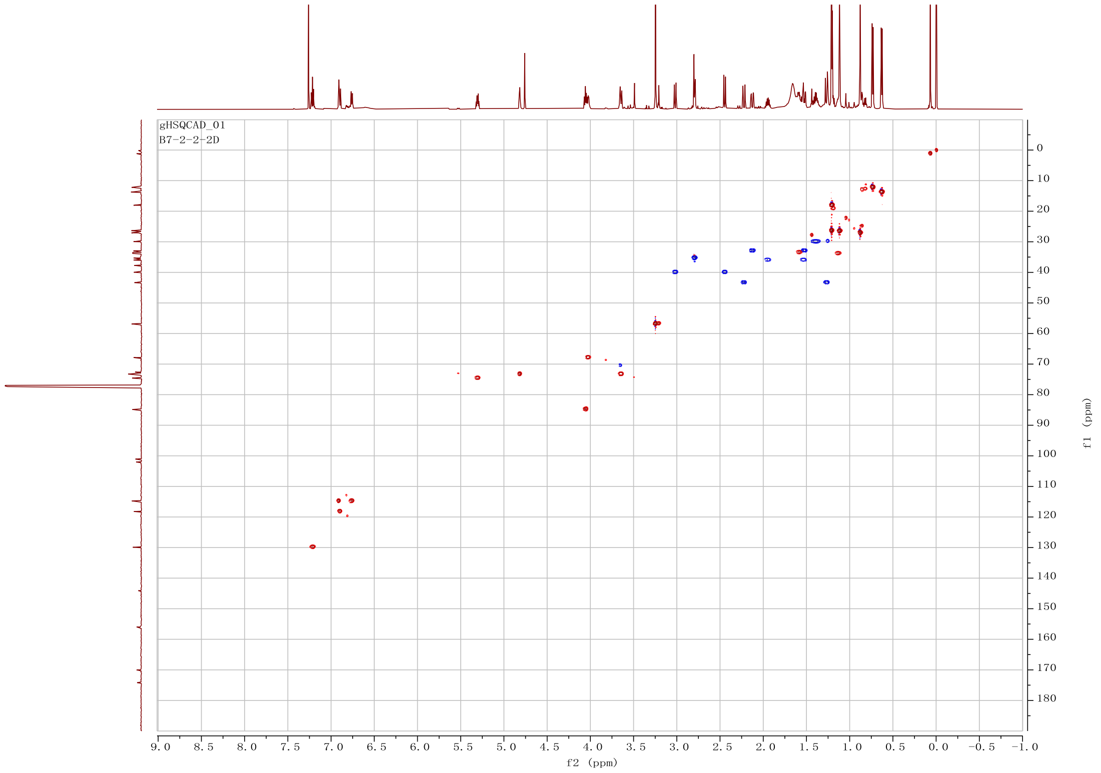

Figure S2.3.5 ^{1}H-^{1}H COSY spectrum of compound **3** in $CDCl_3$.

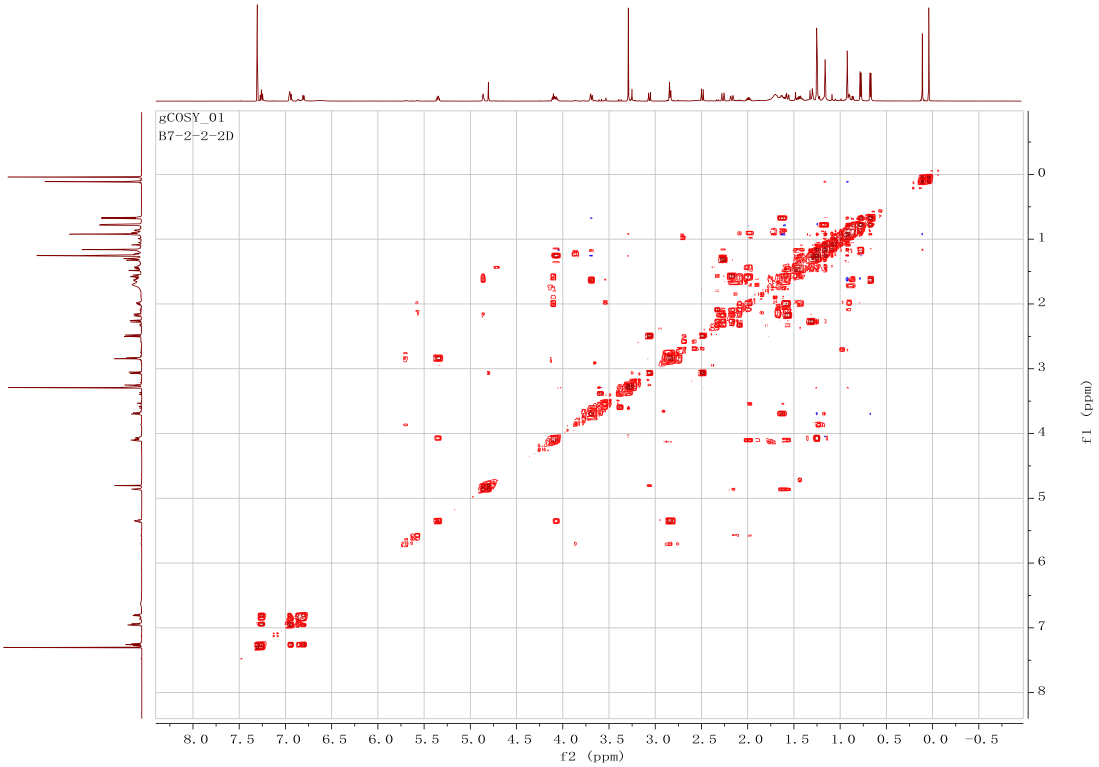

Figure S2.3.6 HMBC spectrum of compound **3** in $CDCl_3$.

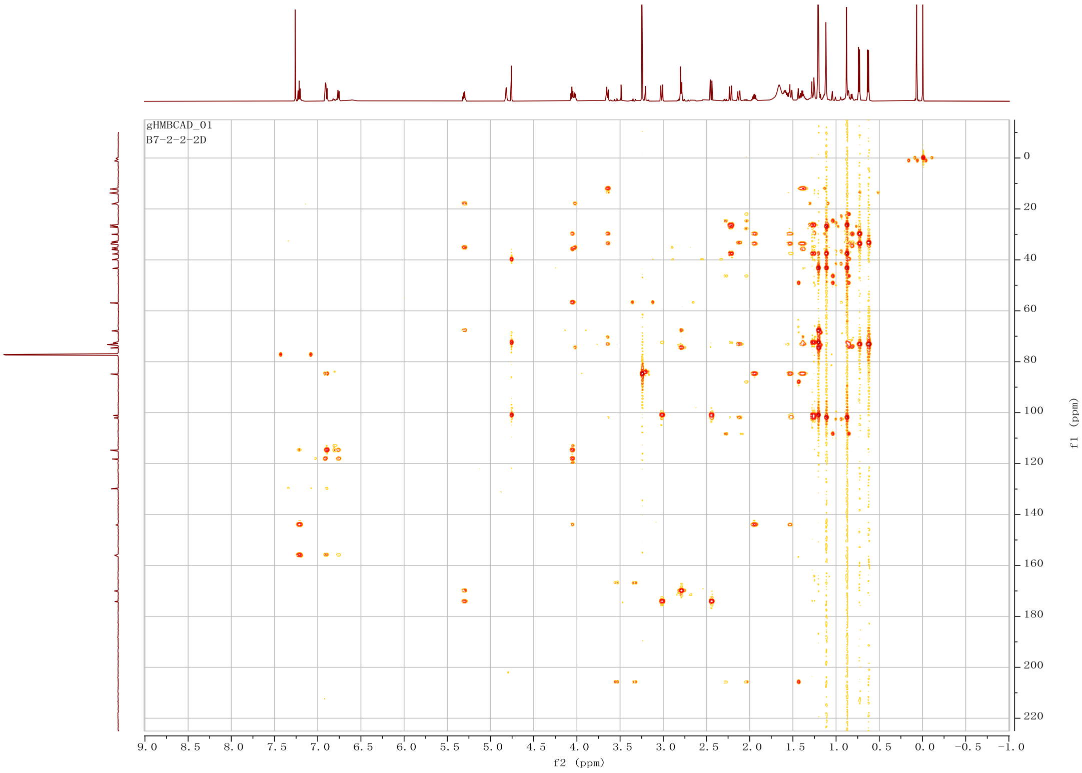

Figure S2.3.7 NOESY spectrum of compound **3** in $CDCl_3$.

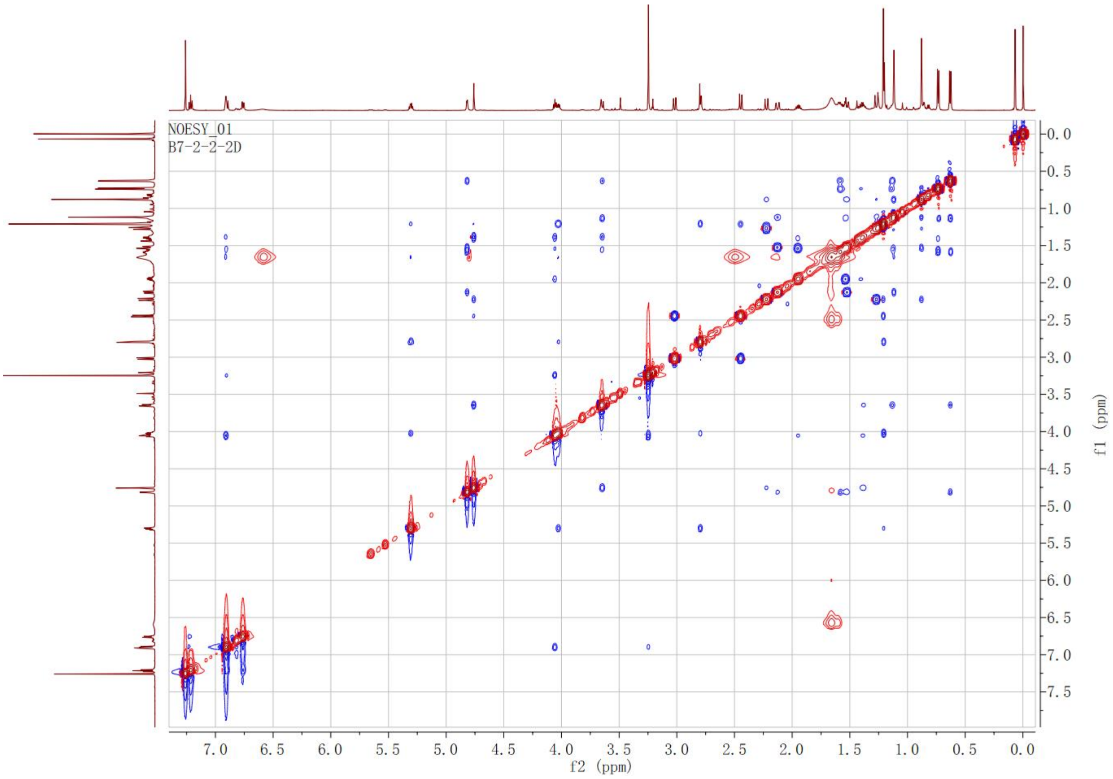

Figure S2.3.8 HRESIMS spectrum of compound **3** in MeOH.

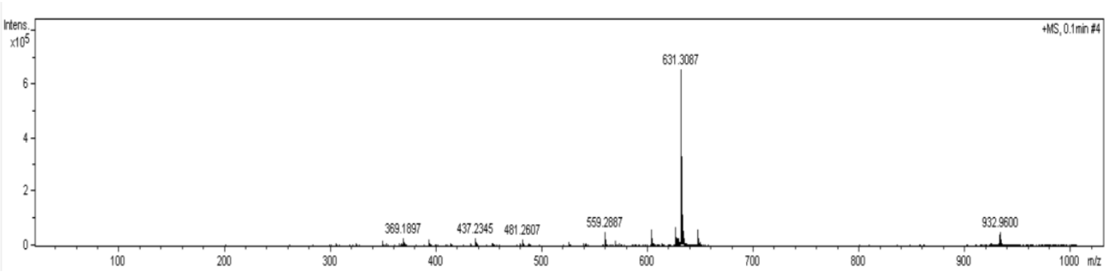

Figure S2.3.9 UV spectrum of **3** in MeOH.

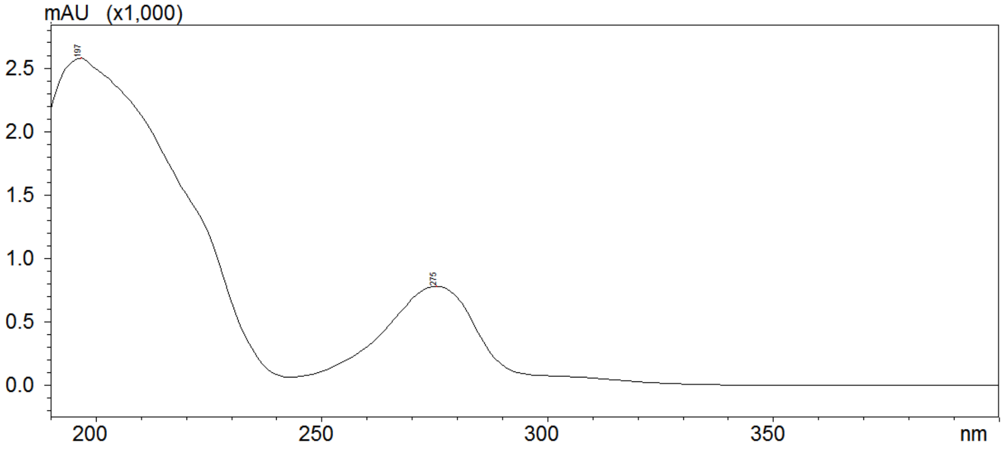

Figure S2.3.10 IR spectrum of **3**.

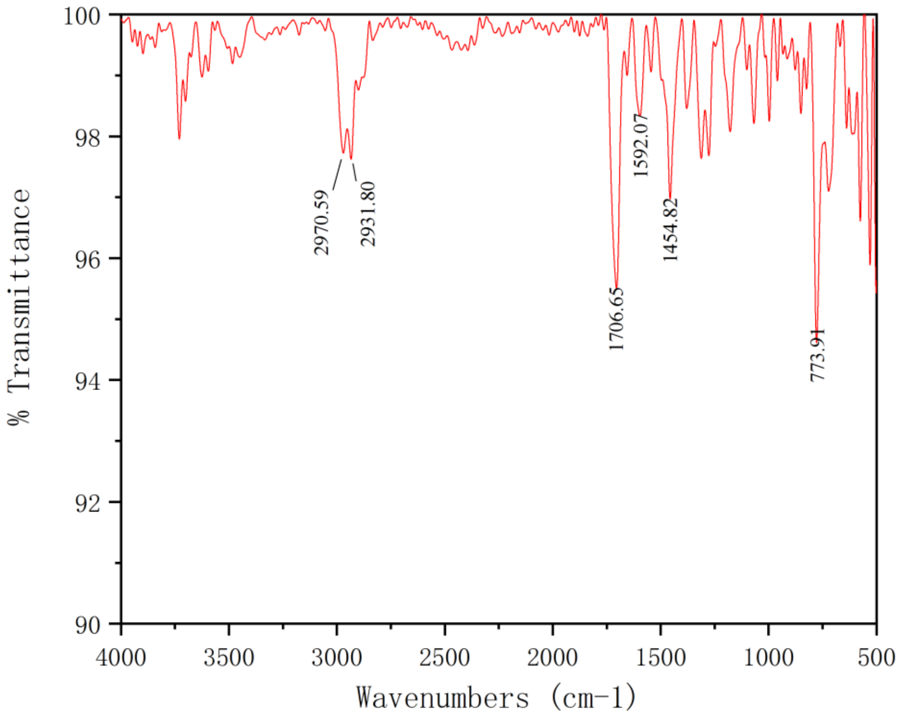

2.4 Spectra Data of compound 4.

Figure S2.4.1 ^{1}H NMR spectrum of compound **4** in $CDCl_3$.

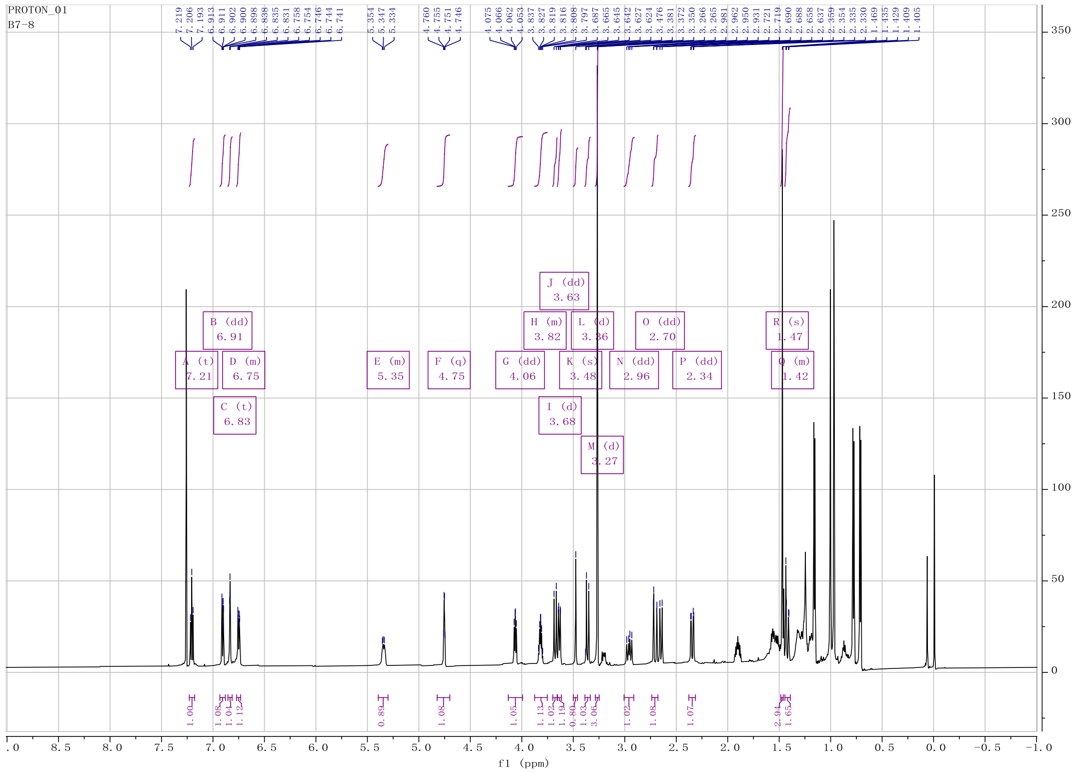

Figure S2.4.2 ^{13}C NMR spectrum of compound **4** in $CDCl_3$.

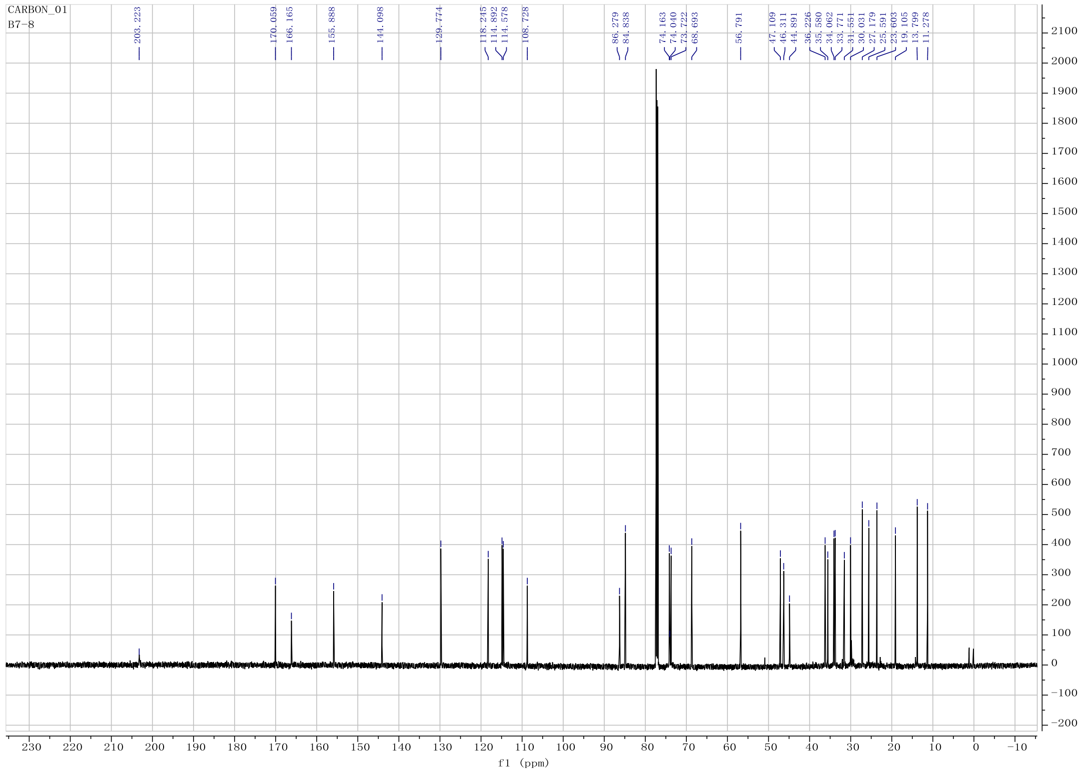

Figure S2.4.3 DEPT spectrum of compound **4** in $CDCl_3$.

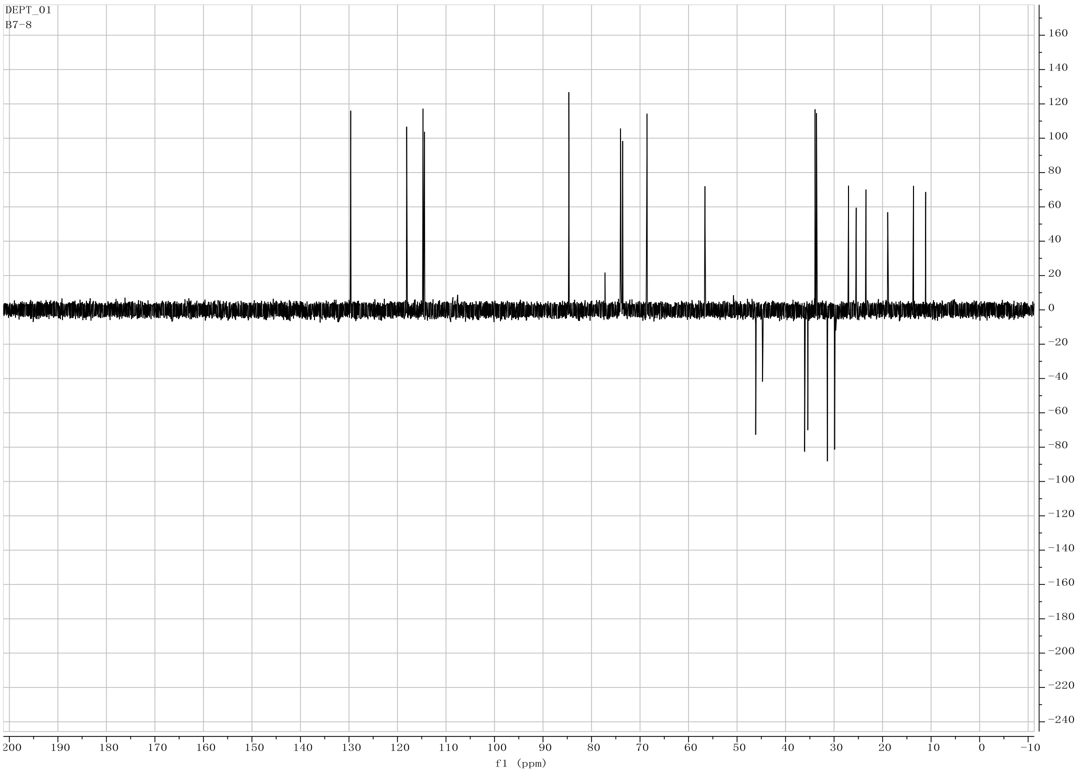

Figure S2.4.4 HSQC spectrum of compound **4** in $CDCl_3$.

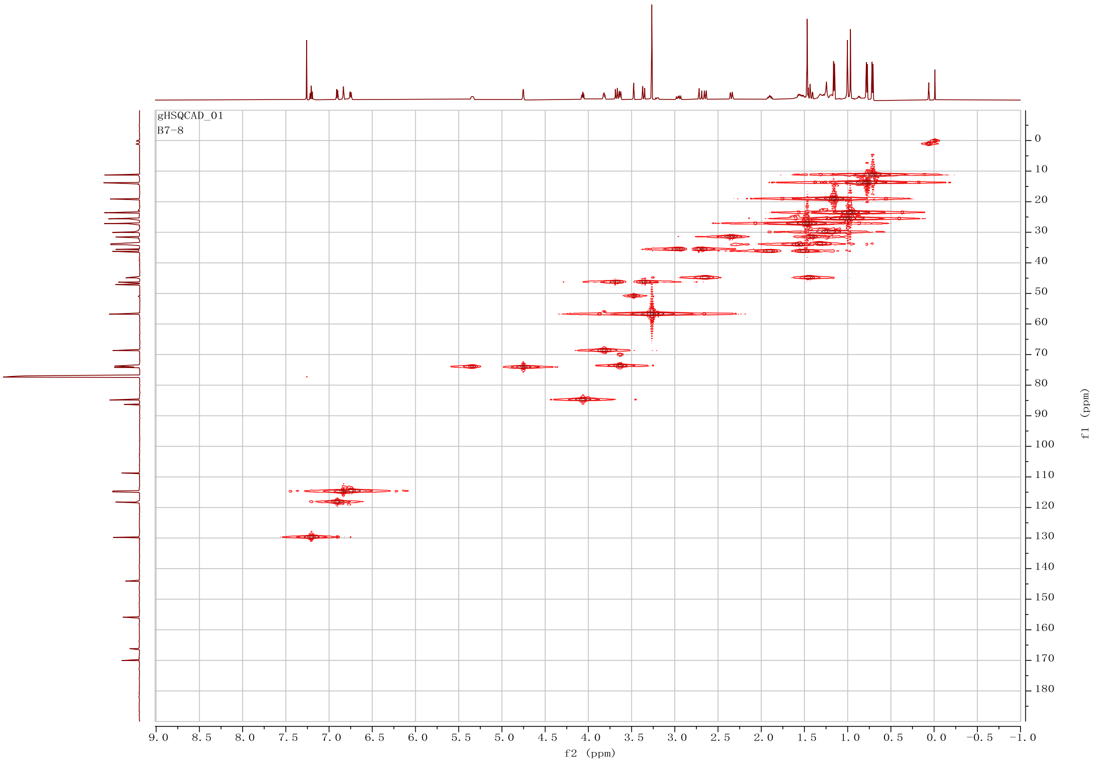

Figure S2.4.5 ^{1}H-^{1}H COSY spectrum of compound **4** in $CDCl_3$.

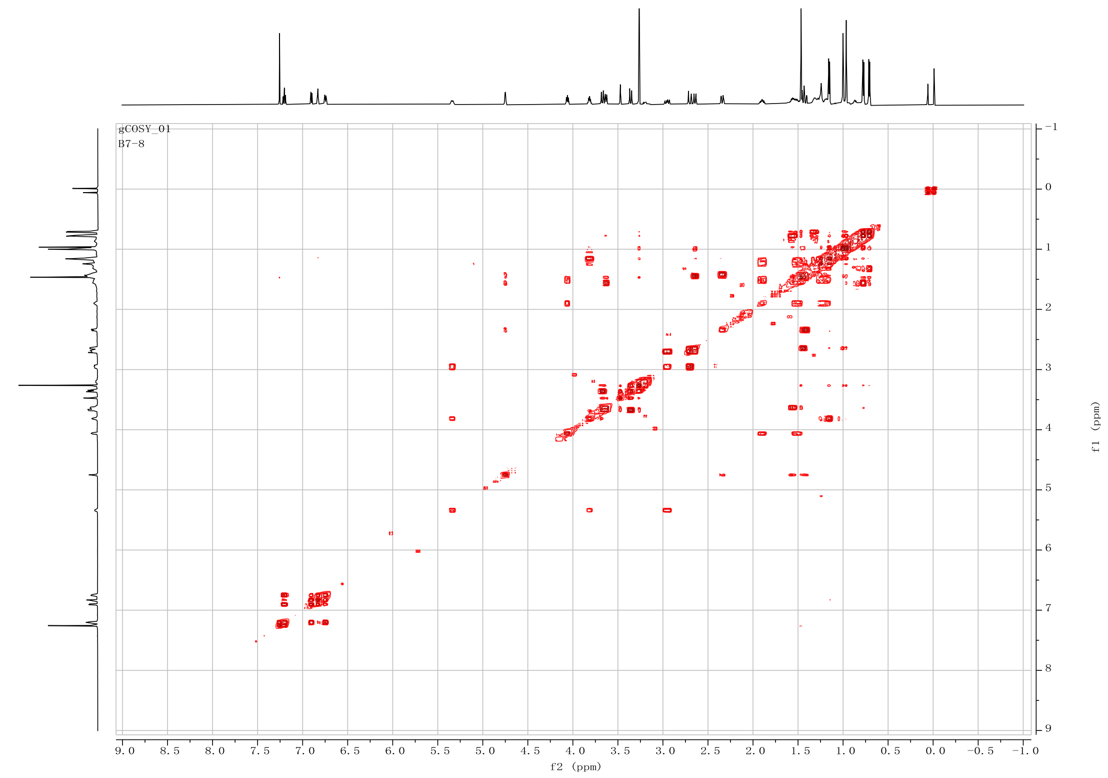

Figure S2.4.6 HMBC spectrum of compound **4** in $CDCl_3$.

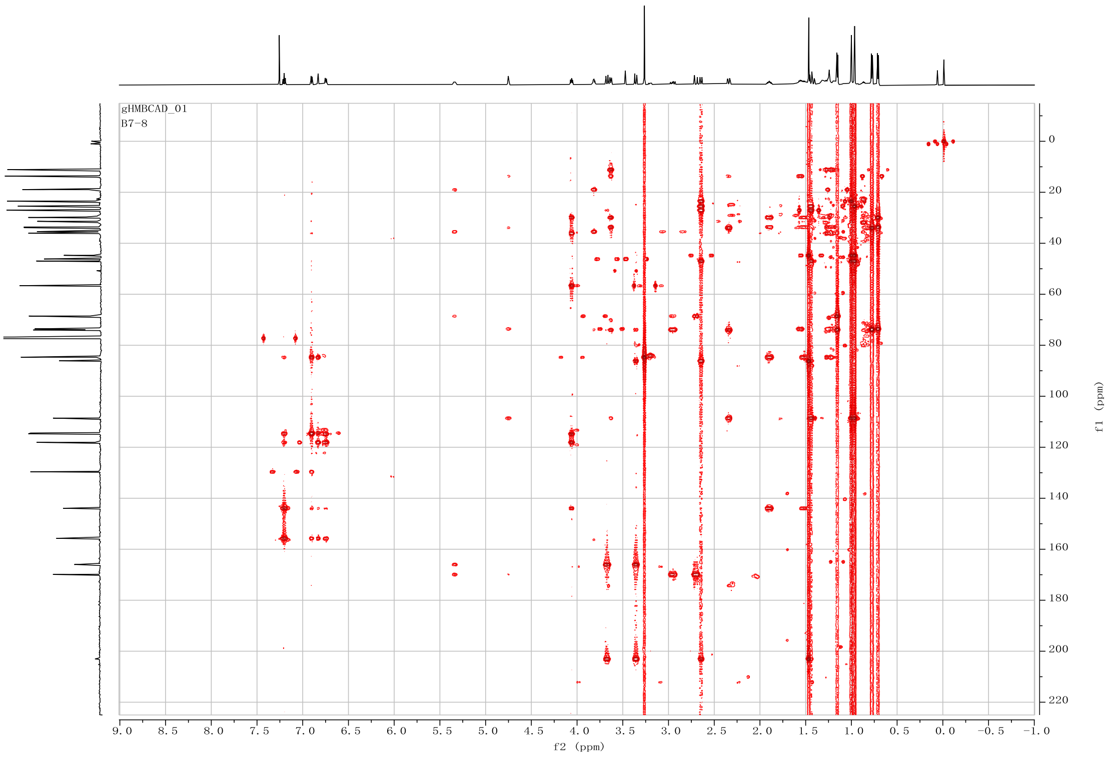

Figure S2.4.7. NOESY spectrum of compound **4** in $CDCl_3$.

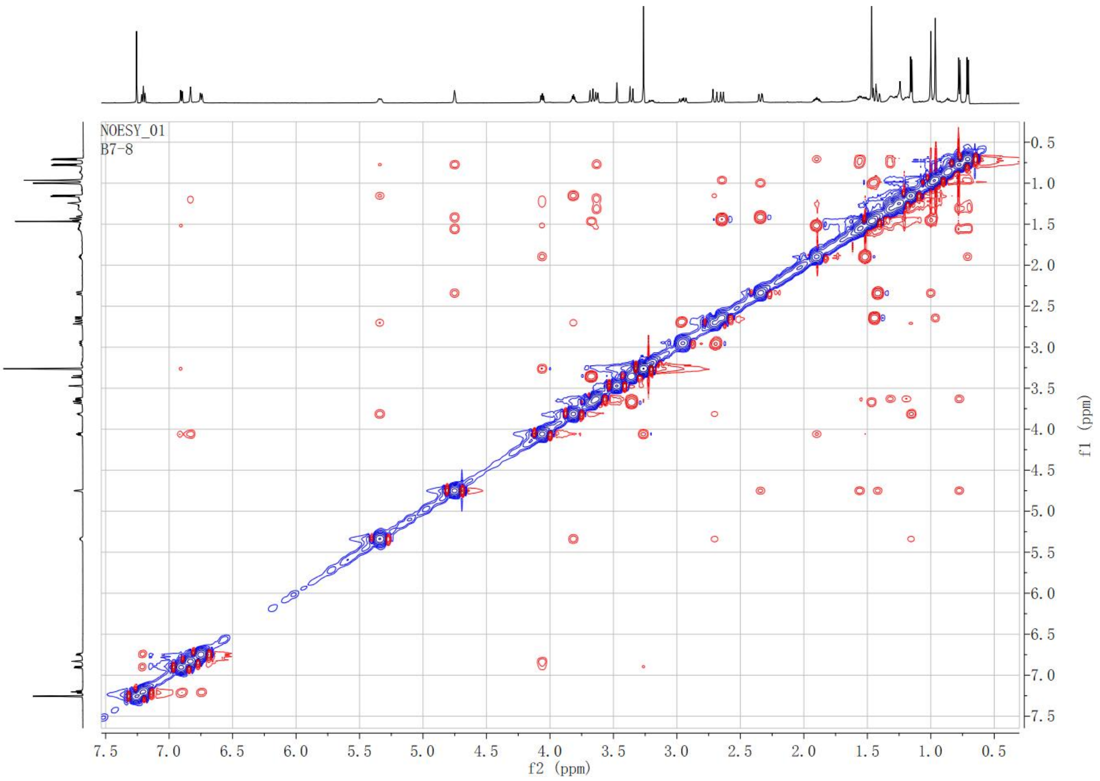

Figure S2.4.8 HRESIMS spectrum of compound **4** in MeOH.

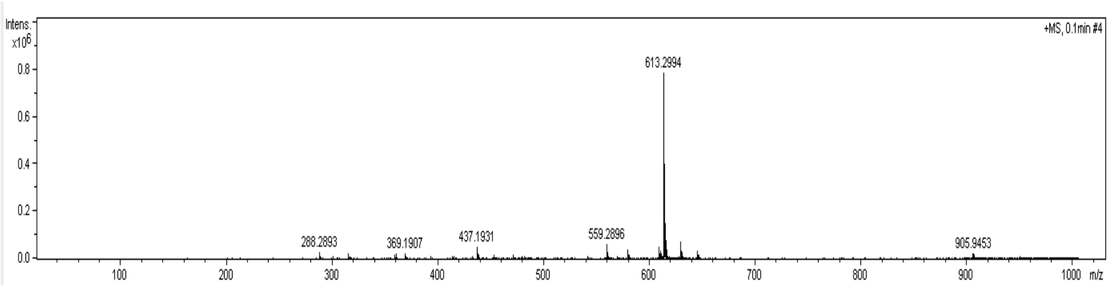

Figure S2.4.9 UV spectrum of **4** in MeOH.

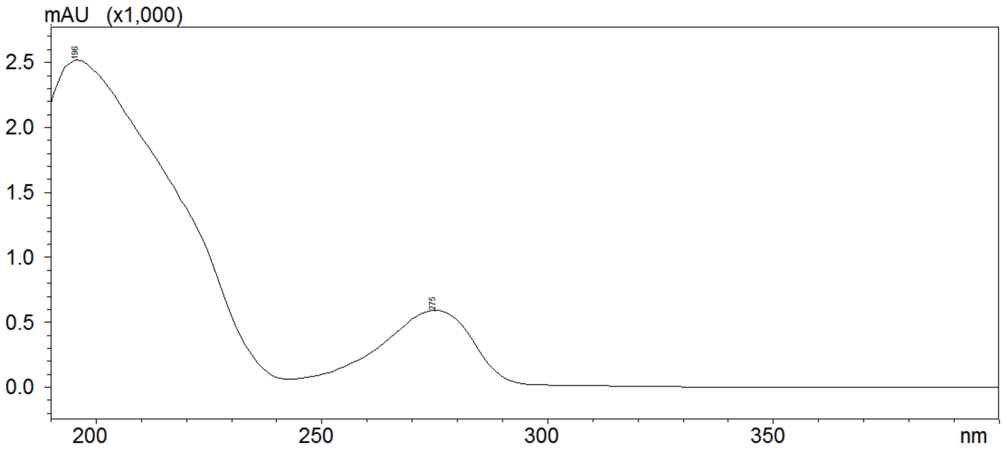

Figure S2.4.10 IR spectrum of **4**.

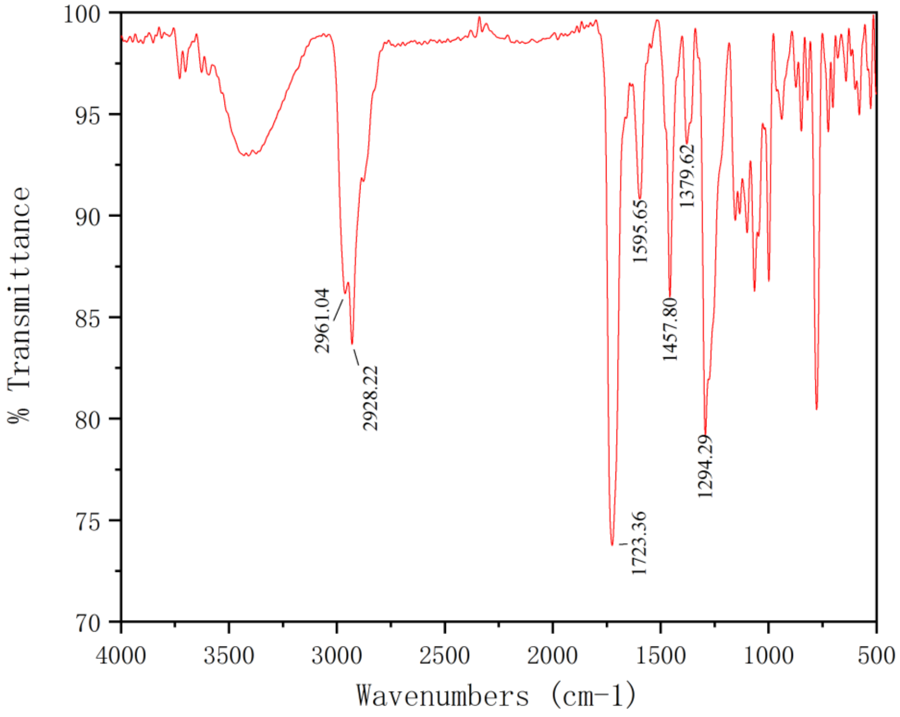

3. NMR Data for debromoaplysiatoxin

Table S3. NMR Data for debromoaplysiatoxin in $CDCl_3$ (δ in ppm, *J* in *Hz*).

Pos.	Debromoaplysiatoxin	
	**δ_H (*J* in Hz)**	**δ_C**
1		169.2
2	a 2.76, d (12.7)<br>b 2.52, d (12.7)	46.9
3		98.8
4	1.84, m	35.7
5	a 1.62, t (13.1)<br>b 1.05, dd (13.1, 3.6)	41.1
6		39.0
7		100.08
8	a 2.68, dd (14.7, 3.0)<br>b 1.71, dd (14.8, 3.6)	33.6
9	5.23, m	73.2
10	1.71, m	35.4
11	3.93, dd (10.9, 2.1)	69.8
12	1.52, m	34.2
13	a 1.31, m<br>b 1.39, m	31.2

14	a 1.97, m<br>b 1.63, m	36.1
15	4.0, dd (8.5, 6.5)	85.8
16		145.9
17	6.92, t (1.1)	114.6
18		158.3
19	6.71, dt (7.9, 1,1)	115.0
20	7.13, t (7.8, 7.8)	129.8
21	6.84, dt (7.9, 1,1)	119.3
22	0.79, d (6.8)	13.6
23	0.71, d (6.9)	13.0
24	0.83, s	26.8
25	0.80, s	16.5
26	0.86, d (6.8)	23.6
27		170.4
28	a 2.92, dd (18.2, 11.1)<br>b 2.87, dd (18.1, 2.8)	34.6
29	5.23, m	74.3
30	4.03, m	67.0
31	1.12, d (6.4)	17.7
15–OCH_3	3.17, s	56.6
